# In vivo intratumoral heterogeneity in a dish: scalable forebrain organoid models of embryonal brain tumors for high‐throughput personalized drug discovery

**DOI:** 10.1002/cac2.70074

**Published:** 2025-11-02

**Authors:** Nicole C. Riedel, Carolin Walter, Flavia W. de Faria, Lea Altendorf, Paula Aust, Carolin Göbel, Archana Verma, Annika Ballast, Ivan Bedzhov, Rajanya Roy, Daniel Münter, Erik Schüftan, Thomas K. Albert, Claudia Rössig, Pascal Johann, Barbara von Zezschwitz, Sarah Sandmann, Julian Varghese, Christian Thomas, Ulrich Schüller, Jan M. Bruder, Kornelius Kerl

**Affiliations:** ^1^ Department of Pediatric Hematology and Oncology University Children's Hospital Münster Münster Germany; ^2^ Institute of Medical Informatics University of Münster Münster Germany; ^3^ Department of Pediatric Hematology and Oncology University Medical Center Hamburg‐Eppendorf Hamburg Germany; ^4^ Research Institute Children's Cancer University Medical Center Hamburg‐Eppendorf Hamburg Germany; ^5^ Embryonic Self‐Organization research group Max Planck Institute for Molecular Biomedicine Münster Germany; ^6^ Pediatrics and Adolescent Medicine, Swabian Children's Cancer Center Experimental pediatrics, University Hospital Augsburg Augsburg Germany; ^7^ Bavarian Cancer Research Center Augsburg Germany; ^8^ Department of Pediatric Hematology and Oncology Charité University Medicine Berlin Germany; ^9^ Institute of Medical Data Science Otto‐von‐Guericke‐University Magdeburg Magdeburg Germany; ^10^ Institute of Neuropathology University Hospital Münster Münster Germany; ^11^ 1Institute of Neuropathology University Medical Center Hamburg‐Eppendorf Hamburg Germany; ^12^ Department of Cell and Developmental Biology Max Planck Institute for Molecular Biomedicine Münster Germany

**Abbreviations**
AB: antibody
ATRT: atypical teratoid and rhabdoid tumor
ATRT‐SHH: atypical teratoid and rhabdoid tumor from the sonic hedgehog (SHH) subgroup hATRT‐SHH: human ATRT from the subtype sonic hedgehog
BABB: benzyl alcohol, benzyl benzoate
C19MC: chromosome 19 microRNA cluster
CNS: central nervous system
ETMR: embryonal tumor with multilayered rosettes
FBO: forebrain organoids
GFP: green fluorescent protein
H&E: Hematoxylin and eosin
hATRT‐SHH: human ATRT‐SHH
hATRT‐SHHGFP+‐FBO: human ATRT‐SHH‐forebrain‐organoid with GFP+ tumor cells
hETMR: human ETMR
hETMRGFP+‐FBO: human ETMR‐forebrain‐organoid with GFP+ tumor cells
hiPSC: human induced pluripotent stem cell
IHC: immunohistochemistry
LIN28A: lin‐28 homolog A
MAP2C: microtubule‐associated protein 2c
Nb: neuroblasts
Nb‐like: neuroblast‐like tumor cells
norm.: normalized
NProg: neuronal progenitors
NProg‐like: neuronal progenitor‐like tumor cells
PFA: paraformaldehyde
prim: primary
RG: radial glia
RG‐like: radial glia‐like tumor cells
ROI: region of interest
scRNA‐seq: single‐cell RNA sequencing
SD: standard deviation
SEM: standard error of the mean
SMARCB1: SWI/SNF‐related matrix‐associated actin‐dependent regulator of chromatin subfamily B member 1
snRNA‐seq: single‐nuclei RNA sequencing
SOX2: SRY‐box transcription factor 2
spheres: tumorspheres (cell lines)
STMN2: stathmin 2
TBO: tumor‐forebrain‐organoid
TME: tumor microenvironment
UMAP: Uniform Manifold Approximation and Projection
undiff: undifferentiated tumor cells
YFP: yellow fluorescent protein

1

Brain tumors are the most prevalent solid tumors in pediatrics, with atypical teratoid and rhabdoid tumor (ATRT) and embryonal tumor with multilayered rosettes (ETMR) presenting particularly poor prognoses.

The development of effective therapies is hampered by the lack of in vitro models that accurately reflect the complex tumor microenvironment (TME) and intratumoral heterogeneity observed in vivo. Traditional monolayer and tumorsphere/tumoroid cultures fail to capture these critical aspects [1], biasing them towards proliferative cell populations that respond differently to drugs than the original tumor. Likewise, in vivo models have fundamental limitations: they lack the primate‐specific chromosome 19 microRNA cluster (C19MC) driver central to ETMR [2], cannot capture human‐specific neurotoxicity, and are impractical for scalable drug screens [1, 3].

To address this, we developed a scalable and reproducible tumor‐forebrain‐organoid (TBO) model for ETMR and ATRT sonic hedgehog (ATRT‐SHH) using a novel coaggregation method, which we characterized histologically and transcriptionally, and applied to drug screening, thereby identifying new candidate therapeutics for ETMR (Supplementary File of Methods). The automated workflow ensures high reproducibility and scalability, enabling the parallel generation of thousands of TBOs for high‐throughput drug screening on tumor and TME.

To integrate central nervous system embryonal tumors into forebrain organoids (FBOs), which recapitulate key developmental trends and contain comparable cell populations found in first‐ and second‐trimester fetal brains, we modified an automated FBO model [4] (Supplementary Figures S1, S3). For this, we employed a coaggregation approach, which involved mixing tumor and human‐induced pluripotent stem cells (hiPSCs), and subsequently allowing their aggregation and joint maturation to form TBOs (Figure 1A). Confocal microscopy of whole‐mount immunostained and cleared organoids revealed a broad and uniform integration of green fluorescent protein (GFP)‐tagged human ETMR and ATRT‐SHH (hETMR and hATRT‐SHH) cells throughout FBOs (Figure 1B). The automated workflow allowed for the parallel generation of highly uniform and reproducible TBOs in 96‐well plates, with low‐standard error of the mean for the GFP signal intensity, indicative for tumor content, across multiple TBOs for both hETMR‐FBO and hATRT‐SHH‐FBO (Figure 1C).

**FIGURE 1:**
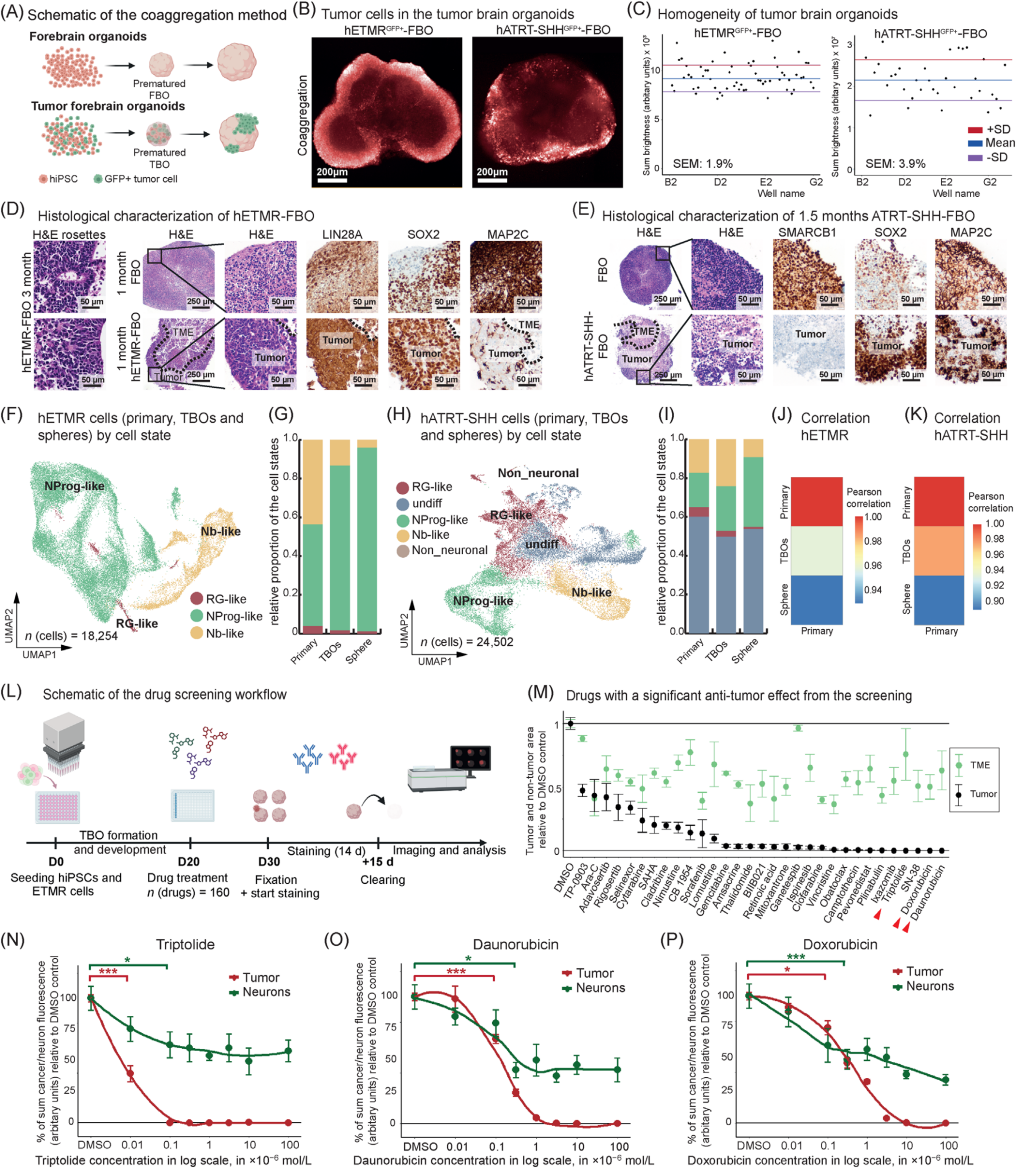
Coaggregation model of forebrain organoids and embryonal brain tumors reflecting intratumoral heterogeneity on histological and transcriptomic levels for high‐throughput personalized drug discovery. (A) Schematic overview of FBO/TBO generation. hiPSCs or a mixture of hiPSCs and tumor cells were seeded, formed an embryoid body and developed an FBO/TBO. Created with BioRender.com. (B) Human GFP‐tagged ETMR and ATRT cells showed a similar growth pattern within the organoids with broad invasion and integration into the TBO. Representative single confocal slices showed whole‐mount immunostained and tissue‐cleared TBOs. (C) Automated staining, imaging, and analysis quantified TBOs in 3D without sectioning. Sum brightness of immunostained cancer cells in hETMRGFP^+^‐TBO or hATRT‐SHHGFP^+^‐TBO. The blue line represents the mean of the summed brightness and the red/violet line the mean ± standard deviation (SD). (D, E) Representative images of immunohistochemical stainings from FBOs and hETMRGFP^+^/hATRT‐SHHGFP^+^‐FBOs generated from serial sections of a FFPE block containing > 40 FBOs show TBOs retaining key protein‐markers. (D) In tumor areas of hETMR‐FBOs, characteristic multilayered rosettes were found. As a hallmark for hETMR, the tumor cells were strongly LIN28A^+^, mainly SOX2^+^, and showed MAP2C expression in a small subset of cells. The control FBOs were mainly LIN28A^+^ and SOX2^+^ and showed high MAP2C expression. (E) As a hallmark for hATRT, the tumor cells were SMARCB1^−^, while FBO cells were SMARCB1^+^, largely SOX2^+^ and showed sparse MAP2C expression. For optimal visualization of histological details in panels (D, E), viewing on a calibrated digital display is recommended. (F) UMAP of the integration of hETMR cells from primary hETMR (prim), 1 month hETMR‐FBOs, and BT‐183 spheres (*n* = 2 primary tumors, *n* = 6 TBO‐samples 1 month; each sample contained 8 physically pooled FBOs, *n* = 2 samples from BT‐183 spheres; colored by cell state. (G) One‐month‐old TBOs more closely recapitulated the cell state distribution of primary hETMR in comparison to tumorspheres. Stacked bar graph shows the relative proportion of the cell states by condition. Colored by cell state as in (F). (H) Integrated clustering of ATRT‐SHH cells from primary ATRT‐SHH (prim; *n* = 4 primary tumors), 1.5‐month ATRT‐SHH‐FBO (*n* = 4 TBO samples, each contained 8 physically pooled FBOs), and 2 ATRT‐SHH sphere cell lines (CHLA‐02 and SHH310‐FHTC; *n* = 2 samples, one per cell line); colored by cell state. (I) Stacked bar graph of the hATRT‐SHH cellular states by condition showed a similar amount of RG‐like and Nb‐like cells in primary hATRT‐SHH and 1.5‐month TBO and a much lower number in tumorspheres, while NProg‐like cells were enriched in tumorspheres. Colored by cell state as in (H). (J) hETMR TBOs resembled primary tumors more than tumorspheres. (K) Likewise, hATRT‐SHH tumors correlated with TBOs more closely than tumorspheres. (L) Schematic overview of the screening workflow for TBOs, starting with the organoid seeding, TBO formation and maturation, followed treatment on day 20 (D20) (*n* = 160 drugs) and fixed on day 30 for subsequent immunostaining, tissue clearing, and high‐content imaging. Created with BioRender.com. (M) Compound screening on hETMR‐FBO revealed over 30 drugs with a significant anti‐tumor effect. Drugs induced different changes in tumor cell (black dots) and TME cell area (grey dots) in TBOs. Error bars indicate the SEM (n ≥ 4 FBOs, 29 planes each, per data point). Ordered by anti‐tumor effect from left (low anti‐tumor activity) to right (high anti‐tumor activity). Red arrows highlight drugs further used for the dose‐response test in N‐P. (N‐P) Dose‐response curves for effects on tumor (GFP^+^) and neuronal cells (MAP2C^+^) after 48h of treatment (*n* = 4 TBOs treated per drug with Daunorubicin (N), Doxorubicin (O) or Triptolide (P)). All values are normalized to the DMSO control. Dots represent the mean of the summed brightness of 1) 647 (tumor) in tumor areas and of 2) 488 (MAP2C^+^) in the whole organoid, summed from all confocal planes. Error bars depict the SEM. Significance indicators are shown for the first significant difference between DMSO control and tumor and neuronal compartments. Two‐sided Wilcoxon rank sum test, * *P* < 0.05, *** *P* < 0.001. Abbreviations: ETMR, embryonal tumor with multilayered rosettes; ATRT‐SHH, atypical teratoid and rhabdoid tumor from the sonic hedgehog subgroup; hETMRGFP^+^‐FBO, human ETMR‐forebrain‐organoid with GFP^+^ tumor cells; hATRT‐SHHGFP^+^‐FBO, human ATRT‐SHH‐forebrain‐organoid with GFP^+^ tumor cells; FBO, forebrain organoid; TBO, tumor brain organoid; hiPSC, human induced pluripotent stem cell; CNS, central nervous system; GFP, green fluorescent protein; AB, antibody; PFA, paraformaldehyde; BABB, benzyl alcohol benzyl benzoate; ROI, region of interest; SD, standard deviation; SEM, standard error of the mean; DMSO, Dimethylsulfoxid; FFPE, formalin‐fixed paraffin‐embedded; H&E, Hematoxylin and eosin; LIN28A, lin‐28 homolog A; SOX2, SRY‐box transcription factor 2; MAP2C, Microtubule‐associated protein 2C; SMARCB1, SWI/SNF‐related matrix‐associated actin‐dependent regulator of chromatin subfamily B member 1; UMAP, Uniform Manifold Approximation and Projection; prim, primary; spheres, tumorspheres (cell lines); RG‐like, radial glia like tumor cells; NProg‐like, neuronal progenitor like tumor cells; Nb‐like, neuroblast like tumor cells; undiff, undifferentiated tumor cells.

To comprehensively characterize TBOs, we employed immunohistochemistry (IHC) to examine the phenotype of hETMR‐ and hATRT‐SHH‐FBOs, with age‐matched FBOs as controls. hETMR tumor areas were identified based on lin‐28 homolog A (LIN28A) positivity, multilayered rosettes, and C19MC alterations, along with GFP immunofluorescence (Figure 1D, Supplementary Figure S4A‐D). Control 1‐month aged FBOs exhibited a predominantly immature phenotype [LIN28A^+^, SRY‐box transcription factor 2 (SOX2)^+^, microtubule‐associated protein 2C (MAP2C)^+^] maturing during culture time (MAP2C^+^, SOX2^−^, LIN28A^−^ at 2‐3 months). hETMR cells consistently displayed positivity for immature progenitor markers (LIN28A^+^, SOX2^+^, and Nestin^+^) across all time points (Figure 1D, Supplementary Figure S4A‐D). Additionally, few hETMR cells exhibited cytoplasmic MAP2C positivity. For hATRT‐SHH, tumor areas were designated based on SWI/SNF‐related matrix‐associated actin‐dependent regulator of chromatin subfamily B member 1 (SMARCB1) negativity in IHC, a hallmark of ATRT, and GFP positivity in immunofluorescence (Figure 1E, Supplementary Figure S4E‐F). Notably, hATRT‐SHH cells were SOX2‐positive and partially MAP2C‐positive (Figure 1E, Supplementary Figure S4E‐F). Both hETMR and hATRT‐SHH cells exhibited a predominantly immature phenotype (SOX2^+^, few MAP2C^+^ cells). Consistent with primary tumors, they showed specific features: C19MC amplification and multilayered rosettes in hETMR and SMARCB1 negativity in hATRT.

Recent investigations have highlighted the substantial intratumoral transcriptional heterogeneity observed in vivo in both ETMR [5] and ATRT [6], a heterogeneity that tumorsphere cell lines do not reflect (Supplementary Figure S5). To assess whether our TBOs better reflect the intratumoral heterogeneity of primary tumor cells compared to tumorspheres, we performed single‐cell RNA sequencing (scRNA‐seq) and integrated our data with publicly available datasets of primary tumors [5, 6, 7]. For ETMR, we classified tumor cells into radial glia‐like (RG‐like), neuronal progenitor‐like (NProg‐like), and neuroblast‐like (Nb‐like) subgroups, with all subpopulations present in hETMR‐FBO (Figure 1F, Supplementary Figure S6A‐B). In contrast, tumorsphere cultures predominantly exhibited NProg‐like cells in hETMR and cycling RG‐like cells in mETMR, indicating a departure from the transcriptional landscape observed in TBOs and primary samples (Figure 1G, Supplementary Figure S6C‐G). For hATRT‐SHH, we employed a signature‐based approach using neuronal signatures from the fetal forebrain atlas [8], classified hATRT‐SHH cells into subgroups resembling fetal radial glia (RG), neuronal progenitors (NProg), and neuroblasts (Nb) and classified the remaining cells as undifferentiated (Figure 1H, Supplementary Figure S6H‐I). Notably, primary and TBO tumor cells displayed a balanced distribution across subpopulations, whereas tumorsphere cultures exhibited a skewed distribution towards the NProg‐like state (Figure 1I). Furthermore, marker genes indicating neuronal differentiation, such as stathmin 2 (*STMN2)*, were notably absent in tumorsphere Nb‐like cells (Supplementary Figure S6J).

Subsequent analysis of cell cycle dynamics revealed that tumorspheres exhibited an accumulation of cycling cells, in contrast to TBOs, which showed similar dynamics as in primary samples (Supplementary Figure S7). Pearson correlation analysis further corroborated the higher correlation between primary tumors and TBO compared to primary tumors and tumorspheres (Figure 1J‐K, Supplementary Figure S8A‐B), underscoring the superior fidelity of TBOs in regaining the transcriptional landscape of primary tumors. Differential expression analysis revealed significant shifts in gene expression patterns between tumorsphere and TBOs (Supplementary Figure S8C‐D), indicating a contextual influence of a neural tissue environment on tumor cell phenotype.

Leveraging the unique capability of our TBO model to simultaneously host tumor and neuronal cells, we established an automated workflow for cell type‐specific drug screening. Following TBO formation and development, drug treatments were initiated on day 20, TBOs were fixed on day 30 for subsequent analysis, followed by whole‐mount immunostaining, tissue clearing, and high‐content imaging (Figure 1L).

As a proof of concept, we identified a therapeutic window for etoposide, a concentration range that maximized anti‐tumor efficacy while minimizing neuronal toxicity, by assessing its effects in both murine ETMR tumor (yellow fluorescent protein; YFP^+^) and neuronal (MAP2C^+^) FBO cells (Supplementary Figure S9). Having established the feasibility of cell type‐specific toxicity screening in TBOs, we next screened a library of > 160 drugs approved by the U.S. Food and Drug Administration (Supplementary Table S1) on hETMR‐FBOs to identify new drug vulnerabilities. Most compounds exerted higher toxicity on tumor than on TME cells (Supplementary Figure S10). Intriguingly, four high‐score drugs from our screen (Figure 1M), doxorubicin, daunorubicin, vincristine, and cytarabine, are currently under investigation in clinical trials as novel therapies against ETMR [9, 10].

Dose‐response curves for three promising candidates with high anti‐tumor activity and low neuronal toxicity – triptolide, doxorubicin, and daunorubicin – validated their anti‐tumor effects (reduction in GFP^+^ cells) and assessed their toxicity to MAP2C^+^ neuronal cells (Figure 1N‐P). Notably, triptolide demonstrated significant anti‐tumor activity with comparatively low neuronal toxicity. The identification of anthracyclines (doxorubicin and daunorubicin), which are under clinical investigation for ETMR [9, 10], was further validated by an independent computational analysis using PERCEPTION (see methods), which predicted high efficacy of anthracyclines against primary ETMR (Supplementary Figure S11). This finding reinforces the predictive validity of our screening platform.

In conclusion, we established ETMR‐ and ATRT‐SHH‐FBO models using a simple, automated coaggregation method that better recapitulated the histological features and transcriptional heterogeneity of primary tumors compared to traditional tumorspheres. Our study validated the approach for cell type‐specific drug screenings and identified anthracyclines and triptolide as potential drugs for ETMR therapy. By combining automation with generation of cancer tissues embedded in healthy tissue and unbiased high‐content analysis in a scalable screening workflow, our work provides proof‐of‐concept for the utility of TBO for exploring anti‐cancer strategies in a complex, near‐native human tissue.

## AUTHOR CONTRIBUTIONS

NCR conducted research, performed experiments, data analysis, and wrote and revised the manuscript. CW, AV, DM and ES conducted bioinformatic data analysis. FF performed experiments, data analysis, held a supervising role, and revised the manuscript. PA performed experiments, data analysis and revised the manuscript. CG, LA, AB, IB, RR and TKA gave direct assistance in conducting experiments and revised the manuscript. CR, CT, PJ, BvZ, SS, JV, and US provided resources and revised the manuscript. JMB contributed to the study design, data analysis, writing and revision of the manuscript, and held a supervising role. KK designed and directed the study, analyzed and interpreted data, revised the manuscript, and provided funding. All authors have read and approved the final version of the manuscript.

## CONFLICT OF INTEREST STATEMENT

Jan M. Bruder is a co‐inventor on the patent application titled “Automated generation and analysis of organoids”, WO2020053257A1. All other authors declare that there is no conflict of interest.

## FUNDING INFORMATION

We thank Daniela Jeising (Department of Pediatric Hematology and Oncology, University Children's Hospital Münster, Münster, Germany) for the processing of scRNA‐seq samples. We thank Dr. Ivan Bedzov and Dr. Kenjiro Adachi (Max Planck Institute for Molecular Biomedicine, Münster, Germany) for sharing their plasmids with us. We thank the Core Facility Genomik (Medical Faculty of Muenster) for the partnership in data sequencing. Bioinformatic analyses for this study were performed in part using the high‐performance computing (HPC) cluster PALMA II at the University of Münster, subsidized by the Deutsche Forschungsgemeinschaft (INST 211/667‐1). Nicole Christin Riedel was supported by funds from the “Medizinerkolleg Münster” (21‐0007) as well as Paula Aust (22‐0028). Kornelius Kerl received funding from Fight Kids Cancer (“EpiRT”), “Deutsche Forschungsgemeinschaft” (KE 2004/4‐1), and Deutsche Kinderkrebshilfe (70114460). Ulrich Schüller was supported by the Fördergemeinschaft Kinderkrebszentrum Hamburg. Figure panels 1A and 1L were created using BioRender.com released under a Creative Commons Attribution‐NonCommercial‐NoDerivs 4.0 International license.

## ETHICS APPROVAL AND CONSENT TO PARTICIPATE

This study considers diversity, equity and inclusion, besides respecting the author's contribution, local resources and regulations for human and animal research. Human tumor tissues were collected after informed consent per protocol approved by the Ethics Committee Münster (2017‐261‐f‐S). Animal experiments were performed following national guidelines and approved by the State Office for Nature, Environment and Consumer Protection, Government of North Rhine‐Westphalia, Germany (81‐02.04.2018.A214; 81‐02.04.2021.A258).

## Supporting information



Supporting Information

Supporting Information

## Data Availability

Raw files of scRNA‐seq data have been deposited at NCBI's Gene Expression Omnibus database (GSE269254, GSE270073 and GSE302654). We used established codes for this study, which are available at the following links: 1) Seurat: https://satijalab.org/seurat/ or https://cloud.r‐project.org/web/packages/Seurat/index.html; 2) CellRanger: https://support.10xgenomics.com/single‐cell‐gene expression/software/pipelines/latest/installation; 3) CONICSmat: https://github.com/diazlab/CONICS.
