# Supplementary material for: In vivo intratumoral heterogeneity in a dish: scalable forebrain organoid models of embryonal brain tumors for high‐throughput personalized drug discovery
Source: Cancer Commun (Lond). 2025 Nov 2;45(12):1670–5. doi: 10.1002/cac2.70074 (PMC12728470; doi:10.1002/cac2.70074)
Supplement: Supplementary file 1 — Supporting Information [file CAC2-45-1670-s002.pdf]

***In vivo* intratumoral heterogeneity in a dish: scalable forebrain organoid models of embryonal brain tumors for high-throughput personalized drug discovery**

Nicole C. Riedel[1], Carolin Walter[1,2], Flavia W. de Faria[1], Lea Altendorf [3,4], Paula Aust[1], Carolin Göbel[3,4], Archana Verma[1], Annika Ballast[1], Ivan Bedhzov[5], Rajanya Roy[1], Daniel Münter[1], Erik Schüftan[1], Thomas K. Albert[1], Claudia Rössig[1], Pascal Johann[6,7], Barbara von Zezschwitz[8], Sarah Sandmann[9], Julian Varghese[9], Christian Thomas[10], Ulrich Schüller[3,4,11], Jan M. Bruder[12], Kornelius Kerl[1*]

[1]Department of Pediatric Hematology and Oncology, University Children's Hospital Münster, Münster, Germany.

[2]Institute of Medical Informatics, University of Münster, Münster, Germany.

[3]Department of Pediatric Hematology and Oncology, University Medical Center Hamburg-Eppendorf, Hamburg, Germany.

[4]Research Institute Children's Cancer, University Medical Center Hamburg-Eppendorf, Hamburg, Germany.

[5]Embryonic Self-Organization research group, Max Planck Institute for Molecular Biomedicine, Münster, Germany.

[6]Pediatrics and Adolescent Medicine, Swabian Children's Cancer Center, Experimental pediatrics, University Hospital Augsburg, Augsburg, Germany.

[7]Bavarian Cancer Research Center, Augsburg, Germany

[8]Department of Pediatric Hematology and Oncology, Charité University Medicine, Berlin, Germany.

[9]Institute of Medical Data Science, Otto-von-Guericke-University Magdeburg, Germany.

[10]Institute of Neuropathology, University Hospital Münster, Münster, Germany.

[11]Institute of Neuropathology, University Medical Center Hamburg-Eppendorf, Hamburg, Germany.

[12]Department of Cell and Developmental Biology, Max Planck Institute for Molecular Biomedicine, Münster, Germany.

[*]Correspondence: Kornelius Kerl, kornelius.kerl@ukmuenster.de

Supplementary Materials and Methods

Cell lines:

The different murine embryonal tumor with multilayered rosette (ETMR) tumorsphere cell lines (*hGFAP-cre::Ctnnb1(ex3)*$^{Fl/+}$*SmoM2*$^{Fl/+}$;YFP^{+}) [1] were provided by the lab of Prof. Dr. Ulrich Schüller (University Medical Center Hamburg-Eppendorf, Hamburg, Germany) (ETMR 1, 2, J) and Prof. Dr. Kornelius Kerl (University Hospital Münster, Münster, Germany) (ETMR T3, T4) [2]. The only available human tumorsphere ETMR cell line worldwide, BT-183 [3], was provided by Prof. Dr. Ulrich Schüller and Dr. Jennifer Chan (University of Calgary, Calgary, Alberta, Canada). The atypical teratoid and rhabdoid tumor from the sonic hedgehog subgroup (ATRT-SHH) cell line SHH-310FHTC was purchased from Olson's Lab (Seattle Children's Hospital, Seattle, WA, United States), and CHLA-02 was purchased from American Type Culture Collection® (Manassas, VA, United States; #CRL-3020). The human-induced pluripotent stem cell line T12 (hiPSC) used for organoid generation was kindly provided by Dr. Jan M. Bruder (Max Planck Institute for Molecular Biomedicine) and characterized in depth by Reinhardt et al. [4]. For a list of all cell lines, their source, and their genetic alterations, please see **Supplementary Table S2**. We regularly tested all cell lines negative for mycoplasma contamination via polymerase chain reaction (PCR).

Cell culture:

All cells were maintained at 37°C and 5% CO_2 in a humidified environment.
hiPSC cell culture: We cultured 300,000 hiPSCs per well on Vitronectin-coated (Thermo Fisher Scientific, Waltham, MA, United States) 6-well plates (Sarstedt, Nümbrecht, Germany) in mTesr Plus medium (STEMCELL Technologies, Vancouver, BC, Canada). The medium was exchanged every other day. For passaging, we detached the cells using 1 mL of Accutase (Thermo Fisher Scientific) per well for 10 min at 37°C and passaged at 1:10-1:20 ratio every 4-6 days until they reached 80%-90% confluence. The detachment reaction was stopped using DMEM/F12 (Thermo Fisher Scientific) supplemented with 0.1% bovine serum albumin (BSA) (Thermo Fisher Scientific) and centrifuged at 220 ×*g* for 2 min at room temperature. After splitting, 300,000 cells were seeded in mTesr Plus medium

supplemented with Rho-Kinase (ROCK) inhibitor Y-27632 (10 µmol/L, Tebubio, Le Perray-en-Yvelines, France).

ETMR cell culture: We cultured human and murine ETMR tumorspheres (each around 200,000 cells per well in > 100 tumorspheres) in suspension culture on Costar ultra-low-attachment 6-well plates (Corning, Corning, New York, United States) in ETMR medium [5] consisting of DMEM/F12 supplemented with 2% B-27 (Thermo Fisher Scientific), 1% penicillin/streptomycin (Thermo Fisher Scientific), basic fibroblast growth factor (bFGF; 20 ng/mL; PeproTech, Cranbury, NJ, United States), epidermal growth factor (EGF; 20 ng/mL; PeproTech), and Heparin (2 µg/mL; STEMCELL Technologies). We exchanged the medium every 2-3 days and split the tumorspheres once per week with Accutase at 37°C when they reached a diameter > 200 µm or developed a dark core. The reaction was stopped after 2 min with 4°C DMEM/F12 and centrifuged at 170 ×*g* for 5 min at room temperature, followed by plating in ETMR medium.

ATRT cell culture: ATRT-SHH tumorspheres (cell lines SHH-310FHTC and CHLA-02) were cultured as suspension cultures in T-25 or T-75 suspension flasks (Sarstedt), with 1 mio cells per T-25 and 3 mio per T-75 flask, in neuronal stem cell (NSC) medium consisting of DMEM/F12 supplemented with 2% B-27, 1% penicillin/streptomycin, 20 ng/mL bFGF, and 20 ng/mL EGF. Tumorspheres were split once per week with Accutase for 5 min at 37°C. To stop the reaction, we used DMEM/F12 and centrifuged at 310 ×*g* for 5 min at room temperature, followed by seeding using NSC medium.

Transfection:

To detect the tumor cells in the forebrain organoids (FBOs), fluorescence genes were transfected into the tumor cell lines and stably integrated via the PiggyBac transposon system [6] The “124_IB_P10_H2B_egfp_IRES_neo” (H2BeGFP) plasmid was kindly provided by Dr. Ivan Bedzov (Max-Planck-Institute for Molecular Biomedicine, Münster, Germany) and “KA2545 PiggyBac” [6] plasmid by Dr. Jan M. Bruder (originally designed and provided by Dr. Kenjiro Adachi, Max-Planck-Institute for Molecular Biomedicine, Münster, Germany). ETMR (BT-183) and ATRT cell lines (CHLA-02 and SHH-310FHTC) were transfected with the H2BeGFP plasmid. To stably transfect tumorsphere lines, tumorspheres were dissociated into single cells

using Accutase 1 h before transfection, and 200,000 cells were seeded per well in a 6-well plate with 2 mL of NSC or ETMR medium. We added a plasmid-free control, missing the H2BeGFP plasmid coding for the antibiotic resistance, to all transfections as a reference during antibiotic selections. For the transfection, 2 µg of plasmid DNA (0.5 µg KA2545 PiggyBac and 1.5 µg H2BeGFP reporter plasmid) were added to 50 µL of Reduced Serum Medium OptiMEM (Thermo Fisher Scientific). In a second tube, 8 µL of FuGene® HD Transfection Reagent (Progema, Fitchburg, WI, United States) was added to 150 µL of OptiMEM, vortexed, and incubated for 5 min at room temperature. Next, DNA/OptiMEM and OptiMEM/Fugene solutions were mixed and vortexed together. After 15 min of incubation, the mixture was vortexed again and added dropwise to the cells.

After 24 h of incubation, we exchanged the medium, and antibiotic selection was initiated 48 h post-transfection with Geneticin (400 µg/mL, Thermo Fisher Scientific) to select cells harboring the H2BeGFP plasmid, which confers neomycin resistance. Antibiotic selection continued until all cells in the non-plasmid control were dead, approximately 48 h.

Organoid culture:

For the generation of automated FBOs, we followed the protocol by Renner et al. [7]. FBO seeding, culture, and feeding were performed using the Biomek FX liquid handling system (v.3.3, Beckman Coulter, Brea, CA, United States), connected to an automated incubator (Cytomat 6001, Thermo Fisher Scientific).

When hiPSCs reached 90%-100% confluence in 2D culture, we manually split the cells using Accutase. All subsequent steps were automated on our Biomek FX liquid handling system. First, 10,000 hiPSCs per well were seeded in 150 µL of seeding medium into 96-well ultra-low-attachment U-bottom plates (Corning). The seeding medium consisted of mTesr Plus medium supplemented with 10 µmol/L ROCK inhibitor Y-27632, and 0.4% w/v polyvinyl alcohol (PVA) (363170, Sigma-Aldrich, St. Louis, MO, United States).
After 24 h, the medium was exchanged by FBO medium [8] consisting of DMEM/F12 supplemented with 20% Knock-Out Serum Replacement (Thermo Fisher Scientific), 1% penicillin/streptomycin, 1% GlutaMAX (Thermo Fisher Scientific), 1% non-essential amino acids (Thermo Fisher Scientific), and 100 µmol/L 2-

Mercaptoethanol (Thermo Fisher Scientific). To induce cortical differentiation, the medium was supplemented with SB-431542 (10 µmol/L; Cay13031, Biomol, Hamburg, Germany) and dorsomorphin (5 µmol/L; Enzo Life Science, Farmingdale, NY, United States) on day 1 and day 4 post-seeding. From day 6 onwards, the medium was exchanged every other day.

From day 6 to day 23, the medium was supplemented with 20 ng/mL epidermal growth factor and 20 ng/mL fibroblast growth factor 2 (both PeproTech). From day 24 to day 45, the medium was supplemented with 20 ng/mL neurotrophin-3 (PeproTech) and 20 ng/mL brain-derived neurotrophic factor (PeproTech). From day 46 on, FBO medium contained no additional growth factors.

Generation of tumor forebrain organoids (TBOs):

To generate TBOs via coaggregation, tumor cells and hiPSCs were split with Accutase as described above. We seeded tumor cells and hiPSCs together in a ratio of 1:10 for hiPSCs/ETMR and 1:20 for hiPSCs/ATRT-SHH, with a total number of 10,000 cells per well using our Biomek FX liquid handling system. Cells were seeded in mTesr Plus medium supplemented with 10 µmol/L ROCK inhibitor Y-27632 and 0.4% w/v PVA in ultra-low-attachment U-bottom plates or Nunc V-bottom plates (Thermo-Fisher). After aggregation, TBOs followed the cortical differentiation protocol [7,8].

Proof-of-concept dose-response experiments using etoposide:

For the proof-of-concept dose-response test, murine ETMR-FBOs were treated for 96 h with varying concentrations of etoposide (E1383; Sigma-Aldrich) starting on day 20 after aggregation. To this end, we performed serial dilutions of Etoposide in dimethylsulfoxide (DMSO; PanReac AppliChem, Darmstadt, Germany) to yield intermediate 200× stock dilutions ready for addition to the final experimental organoid plates. On day 20 of TBO differentiation, we diluted the intermediate stocks to 1:200 in FBO-medium using our Biomek FX liquid handling system and treated 8 TBOs per concentration/condition (final concentrations: 0.1, 0.3, 1, 3, 10, 30, 100 µmol/L). We renewed the medium, including etoposide, after 48 h. After 96 h, we reverted to etoposide-free FBO medium. TBOs were fixed for whole-mount immunostaining on day 30 of differentiation.

Automated compound screening:

For compound screening, we used a customized compound library consisting of 60 drugs selected from the Cambridge Cancer Library (Selleckchem) and 100 drugs from the Compound Management and Screening Center (Max-Planck-Institute of Molecular Physiology, Dortmund, Germany). All compounds are listed in **Supplementary Table S1**. Each experimental plate included solvent-only (DMSO) controls, no-primary antibody controls (TBOs stained with secondary but not primary antibody), and no-cancer controls (FBOs without tumor cells). We diluted the drugs, stored on a 384-well plate (Greiner, Kremsmünster, Austria), with our Biomek FX liquid handling system 1:1000 in pre-warmed FBO medium (1 µL of drug in 999 µL medium) from a 10 mmol/L stock (with some exceptions at 2 or 8 mmol/L due to solubility constraints) using Biomek Tips P50 (B85888; Beckman Coulter). Using Biomek Tips P250 (717252; Beckman Coulter), the drug solution was mixed, and TBOs (one per well) were treated with 150 µL per well of the drug solution during a standard automated medium exchange. After 48 h, a medium exchange was performed with a drug-free FBO medium, followed by medium changes every other day. TBOs were fixed for subsequent whole-mount immunostaining at day 30.

Dose-response validation experiments for daunorubicin, doxorubicin, and triptolide:

As a follow-up for the primary screening results above, we conducted dose-response experiments for daunorubicin (#HY-13062; MedChemExpress, Monmouth Junction, New Jersey, USA), doxorubicin (#HY-15142; MedChemExpress) and triptolide (#HY-32735; MedChemExpress). First, we dissolved the drugs in DMSO to prepare 50 mmol/L stocks. These were serially diluted to create 500× intermediate stocks and manually loaded on a 384-well plate. On day 20 of differentiation, four TBOs per condition were treated with intermediate stocks diluted 1:500 in FBO medium using a liquid handling system (1 µL of drug in 499 µL of medium), resulting in final concentrations of 0.01, 0.1, 0.3, 1, 3, 10, and 100 µmol/L. After 48 h, we exchanged the medium for drug-free FBO medium, followed by automated medium changes every other day. We fixed TBOs for later whole-mount immunostaining on day 30 of differentiation.

Histology, Immunohistochemistry and fluorescence in situ hybridization (FISH):

For histological staining, we used formalin-fixed paraffin-embedded (FFPE) sections from FBOs and TBOs. Each block contained over 40 TBO/FBOs, and serial sections were cut at 2 μm thickness. Due to cutting, only part of the input organoids can be seen on one slide. For each FFPE block, we performed IHC staining. The hematoxylin and eosin (H&E) staining was performed according to established protocols [9]. We used the ultraView, OptiView, or iView DAB Detection Kit on a Ventana Benchmark xt System (Roche Diagnostics, Basel, Switzerland) according to the manufacturer's instructions. The list of all antibodies used can be found in **Supplementary Table S3**. FISH was performed using the C19MC/TPM4 FISH Probe Kit (CT-PAC03, Cytotest Inc., Rockville, MD, USA) according to the manufacturer's instructions.

Quantification of IHC:

For the immunostaining quantification, representative images were taken at 4× magnification with a brightfield microscope (BX43, Olympus Corporation, Tokyo, Japan) using the Olympus cellSens software (Olympus Corporation) to capture a whole organoid. In addition, the GNU Image Manipulation Program (GIMP; https://www.gimp.org/; version: 2.10.38) was used for white balance correction of brightfield images. Quantification was performed using ImageJ (1.53c, Java 1.8.0_172; https://imagej.net/ij/). At first, color deconvolution was applied using ImageJ's *H DAB* vector. The resulting image (*Colour 2*) was cropped by selecting only the tumor area. Positive areas were measured using the Image-J-Macro below with different thresholds (Th) for different stainings (GFAP and Synaptophysin: Th = 150; Nestin: Th = 175; OLIG2: Th = 100). The percentage of positive areas was calculated by normalization to the total organoid area. Three organoids were quantified for each condition. Statistical analyses were performed with a one-sided Wilcoxon rank sum test.

Image-J-Macro:

```
imageTitle=getTitle();// run("Set Measurements...", "area area_fraction limit display
redirect=None decimal=3"); run("8-bit"); setAutoThreshold("Default dark");
//run("Threshold..."); setThreshold(0, Th); setOption("BlackBackground", true);
run("Convert to Mask"); run("Measure");
```

Immunofluorescence of FFPE-embedded TBOs:

FFPE tissue slides were deparaffinized in Xylol (TH.GEYER, Renningen, Germany) 2 times for 10 min each. This was followed by a descending ethanol series: twice for

5 min in 100% ethanol, followed by once for 5 min in each of 95%, 90%, 80%, 70%, and 50% ethanol. For antigen retrieval, the slides were heated in 10 mmol/L trisodium citrate dehydrate (Carl ROTH, Karlsruhe, Germany) in ddH_2O, pH 6.0, using a streamer for 20 min. The slides remained in the buffer to cool down for 30 min. Next, the slides were rinsed in ddH_2O for 5 min, then washed three times for 5 min each in 0.3% Triton X-100 (Carl ROTH) in phosphate-buffered saline (PBS-T), and blocked with 5% normal goat serum (NGS; Merck, Darmstadt, Deutschland) in PBS-T for 1 h at room temperature. After blocking, the slides were incubated with the primary antibody (1:500 anti-GFP: ab13970, Abcam, Cambridge, United Kingdom), dissolved in NGS + PBS-T, at 4°C overnight. The next day, slides were washed three times with PBS-T (for 5 min each) and incubated with secondary antibodies (Alexa Fluor 647, 1:1000; Thermo Fisher Scientific) and 4',6-diamidin-2-phenylindol (DAPI; 1 μg/mL; Sigma-Aldrich), dissolved in PBS + 0.3% NGS for 1 h at room temperature in a humidified chamber. This was followed by a final washing step with PBS (3 times for 5 min), and, finally, the slides were mounted with Mowiol 4-88 (Carl ROTH; solution prepared according to manufacturer's instructions). Imaging was performed using a Nikon Ti2 microscope (Nikon, Tokyo, Japan) and Nikon Imaging Software NIS-Elements (Nikon, version 5.11.03 (Build 1373) 64-bit). Native 16-bit TIFFs from the acquisition were imported into Fiji/ImageJ, split into their respective channels using the "stack to images" function. They were then merged and pseudocolored using the "merge channels" function. Here, the DAPI channel was assigned to the blue channel, and the 647 nm channel labeling the primary/secondary Alexafluor 647 against GFP was assigned to the green channel. Individual channel contrast was adjusted using the "maximum" and "minimum" sliders of the contrast module on a per channel basis to optimize their respective white and black points. Finally, images were exported as 8-bit RGB images.

Whole-mount immunostaining and benzyl benzoate and benzyl aldehyde-based tissue clearing:

To perform automated whole-mount immunostaining, we followed the automated whole-mount immunostaining and tissue clearing protocol outlined by Renner et al. [10] with minor modifications. All liquid handling steps of this protocol were performed automated using the Biomek FX liquid handling system. TBOs were fixed in 4% paraformaldehyde (PFA; Thermo Fisher Scientific) for 15 min and washed with

PBS three times. All antibodies were diluted in a blocking solution consisting of 6% BSA in PBS, 0.5% Triton-X-100 (Sigma-Aldrich), and 0.1% sodium azide (Sigma-Aldrich). TBOs were incubated for 6 days at 37°C in a humidified atmosphere with the primary antibodies: 1:500 anti-GFP; 1:500 anti- Microtubule-associated protein 2 (MAP2): ab32454, Abcam; 1:1000 anti-MAP2: MAB3418, Sigma-Aldrich. Following primary antibody incubation, TBOs were incubated with secondary antibodies and DAPI for another 6 days: Alexa Fluor 647 donkey/goat anti-chicken, Alexa Fluor 488 donkey anti-rabbit, Alexa Fluor 647 donkey anti-mouse; All secondary antibodies diluted 1:1000, Thermo Fisher Scientific and DAPI (0.5 µg/mL; Sigma-Aldrich). The antibody solution was refreshed every other day. Post-incubation, samples were washed five times with 0.1% Triton X-100 in PBS and incubated at 37°C in the dark. Each plate included no-primary antibody controls (TBOs processed without the primary antibody) and no-cancer controls (FBOs without tumor cells but subjected to full staining).

For tissue clearing, samples were dehydrated through a methanol (Carl ROTH) dilution series in distilled water: 25%, 50%, 75%, 90%, 100%; 60 min each per concentration with one exchange. Next, we transferred the samples with wide bore tips to solvent-resistant cyclo-olefin-coated 96-well plates ("Screenstar", Greiner) and incubated the samples for 120 min in 1:1 v/v methanol/BABB, with one solution refresh after 60 min. Finally, samples were incubated in pure BABB (1:1 v/v benzyl benzoate and benzyl aldehyde, both Sigma-Aldrich) and stored in the dark at 4°C until imaging. To ensure uniform aggregate positioning for imaging, the plate was positioned at a 30° incline.

Confocal high-content imaging and image analysis:

We performed confocal imaging on an Operetta high-content imager (Perkin Elmer) equipped with a 10× long working distance lens. We determined suitable exposure times for each channel using no-primary antibody controls and no-cancer controls to assess the background level of staining on each plate. We applied the resulting parameters equally for all samples from one experiment.

We analyzed all images in Columbus version 2.6.0 (Perkin Elmer) following the general protocol of Renner et al. [11]. For dose-response experiments with etoposide, doxorubicin, daunorubicin, and triptolide, we used maximum projections of all

confocal planes for analysis. We were careful not to oversaturate the imaging sensor and work within the bit-depth of the operetta files, also when collapsing Z-stacks via Maximum projections for analysis. This allowed us to maintain a quantitative workflow while minimizing the number of images for segmentation and quantification. For the ETMR/ATRT homogeneity tests and compound screens, single optical confocal planes were quantified, and data were summed across Z-planes after segmentation and quantification. Specifically, for the ETMR homogeneity screen, we used 17 planes with an inter-plane distance of 35 μm; for the ATRT homogeneity screen, we used 21 planes with an inter-plane distance of 29 μm; for the compound screen, we used 29 confocal planes with an inter-plane distance of 21 μm. The number of planes and inter-plane distance depended on the maximum size and Z position of TBOs we encountered in each experiment and was adjusted to minimize acquisition time while sampling the full TBO height.

We applied automatic flatfield correction and used a combination of DAPI, 647, and, if applicable, 488 channels to detect organoids. Organoids were identified by summing the channels, applying a Gaussian blur (10 pixels), and using a brightness threshold of 0.05 with the "Find Image Region" function of Columbus. To exclude sensor noise, hot pixels, dust, and other image artifacts, bona fide organoid regions were kept if they exceeded 5000 square pixels per consecutive area and field of view.

Tumor cells expressed GFP (human ETMR and human ATRT-SHH) or yellow fluorescent protein (YFP) (murine ETMR), and native fluorescence was quenched using BABB clearing. GFP/YFP signals were detected with anti-GFP/YFP primary antibodies and amplified with an Alexa Fluor 647 secondary antibody (see details above). These Alexa Fluor fluorophores continued fluorescing under BABB clearing conditions, and their long wavelength provided superior tissue penetration and cancer cell detection compared to native GFP or YFP fluorophores. Further downstream analysis differed slightly between experiments and is described below.

Etoposide proof of concept: Murine ETMR tumor cells in the 647 channel within the identified organoid area were detected using the "Find Image Region" function with a threshold of 0.40. Objects were classified as YFP$^+$ cancer areas if they exceeded 500 arbitrary brightness units and 10 square pixels. In these cancer areas, the sum brightness was measured. MAP2 staining was assessed by measuring the total

brightness in the MAP2-488 channel for the identified organoid region, summing the results across all planes and fields of view per TBO.

Homogeneity, compound, and drug validation: Human ETMR or ATRT-SHH H2BeGFP-transfected tumor cells fluorescing in the 647 channel and located in the previously segmented organoid area were identified using the “Find nuclei function”. Nuclei were selected based on brightness thresholds determined individually for each experiment using non-tumor control samples on the same plate. The area of the whole organoid, cancer area and sum brightness of cancer area were measured in all experiments, and if applicable also MAP2 brightness. For subsequent analysis, whole-organoid area, cancer area and brightness across the segmented organoid area were summed for all fields of view and all Z-planes.

Further analysis was performed in Microsoft Excel 2016 (Microsoft Corporation, Redmond, WA, USA) and visualized with the R package ggplot2 [12]. Therefore, raw output files from Columbus (version 2.6.0; Perkin Elmer) were imported into Excel, where the drugs were annotated to their respective wells. We excluded data from further analysis from organoids that were not completely imaged or were lost or damaged during staining and clearing, as well as data from wells with dust particles in or around the organoids. With the data from all fully-imaged and intact organoids, we calculated the tumor microenvironment (TME) area by subtracting the tumor area (647^+) from the whole-organoid area ($DAPI^+$). As a next step, all values were normalized to the medium of the 64 DMSO controls for the respective tumor/TME or whole-organoid area. After normalization, the mean and the standard error were calculated per drug. These values were then used as input to generate graphs using ggplot2 [12] in R version 4.2.2 [13].

Statistical tests for imaging data:

We used different statistical tests using R version 4.2.2 [13] to analyze the imaging data. We considered *P*-values as significant if they were < 0.05. For comparison of the dose-response data, we used a two-sided Wilcoxon rank sum test. For the screening data, we used multiple unpaired t-tests followed by correction for multiple testing with the Benjamini & Hochberg method (**Supplementary Table S4**). Drugs were identified as potential hits, when the treatment resulted in a significant change in the tumor area, but a non-significant change was observed in the TME.

Human sample acquisition

We collected all fresh and fixed ETMR and ATRT-SHH samples after informed consent per protocols approved by the Ethics Committee Münster (2017-261-f-S) in 2021, diagnosed via standard clinical pipelines, including immunohistochemistry, 850k methylation array, and molecular inversion probe copy number variation (CNV) showing C19MC amplification. Clinical data of previously unpublished samples are detailed in **Supplementary Table S5**.

Genetically engineered mouse models

All animal procedures were approved and performed according to local guidelines (State Agency for Nature, Environment and Climate, Government of North Rhine-Westphalia, Germany; reference numbers 81-02.04.2018.A214; 81-02.04.2021.A258). The animals were raised and maintained at the Central Animal Experimentation Facility of the University of Münster (Münster, Germany). All used mouse strains *SmoM2*$^{fl/fl}$ [14], *hGFAP-cre* [15], *Ctnnb1(ex3)*$^{fl/fl}$ [16] and a *hGFAP-cre::Ctnnb1*$^{(ex3)fl/+}$*SmoM2*$^{fl/+}$ [1] strain (with constitutive expression of a YFP reporter gene under the SmoM2 promoter) were previously generated and characterized. To generate parenteral *SmoM2*$^{fl/+}$*Ctnnb1(ex3)*$^{fl/+}$ strain, which does not exhibit a phenotype, we crossed *SmoM2*$^{fl/fl}$ with *Ctnnb1(ex3)*$^{fl/fl}$ mice. Crossing this strain then with *hGFAP-cre* mice, we obtained *hGFAP-cre::Ctnnb1*$^{(ex3)fl/+}$*SmoM2*$^{fl/+}$ mice, which developed an ETMR phenotype via upregulation of Wnt and Shh pathways at an embryonal age [1], which is lethal until postnatal day 1. We monitored the pregnant mice from this crossing daily for distress signs (lethargy, weakness, pain). To obtain ETMR tumors, the pregnant mice underwent cesarean section under analgesia and sedation at E18.5 post-coitum (post-coital plug observation was considered as day 0.5) and were sacrificed by cervical dislocation. For the tumor isolation from the embryos, we decapitated them and Cre^{+} forebrains were used for further experiments. We performed the genotyping of the mice via polymerase chain reaction (30 cycles with each 30 s at 95°C, 45 s at 60°C and 1 min at 72°C) of tail or cerebellum DNA for *Cre, Ctnnb1* and *SmoM2* using the following primers: *Cre*- Forward: 5´-TCCGGGCTGCCACGACCAA-3`; *Cre*-Reverse: 5´-GGCGCGGCAACACCATTTT-3`; *Ctnnb1*- Forward: 5´-CGTGGACAATGGCTACTCAA-3; *Ctnnb1*-Reverse: 5`-

TGTCCAACTCCATCAGGTCA-3`; *SmoM2*-Forward: 5`-GGAGCGGGAGAAATGGATATG -3`; *SmoM2*-Reverse: 5`-CGTGATCTGCAACTCCAGTC-3`.

Dissociation of fresh human and murine ETMR tumors for single-cell RNA sequencing (scRNA-seq)

We used a fresh biopsy of human ETMR and fresh murine forebrains of *hGFAP-cre::Ctnnb1*$^{(ex3)fl/+}$*SmoM2*$^{fl/+}$ embryos at a gestational age of embryonal day 18.5 for single-cell dissociation. We generated the single cell solution via a combination of mechanical and enzymatic digestion, starting with cutting the tumors into 1-2 mm^3 pieces, followed by digestion for 30 min at 37°C in papain-solution consisting of 20 units of papain (Worthington, Columbus, OH, United States) dissolved in pre-warmed DMEM/F12 with 32.2 µg/mL DNase (Worthington). We passed the cells through a 40 µm cell strainer (Corning) and depleted red blood cells using Ammonium-Chloride-Potassium lysis buffer (Thermo Fisher Scientific) according to the manufacturer´s instructions. Via flow cytometry with a BD FACSAria II Cellsorter (BD Biosciences, Franklin Lakes, NJ, United States), we sorted 10,000 vital cells from the fresh murine and cryopreserved human cell suspensions using 7-Aminoactinomycin D (7-AAD, eBioscience, San Diego, CA, United States) staining and used them as an input for scRNA-seq.

Dissociation of tumorspheres and TBOs for scRNA-seq:

Cell lines: ATRT-SHH and BT-183 tumorspheres were dissociated using Accutase as described above.

Organoids: To get an appropriate cell number, we pooled 8 FBOs/TBOs to form one pooled scRNA-seq sample. For FBO and TBO samples, we used a Papain-based dissociation protocol. To this end, we incubated 8 organoids each in 2 mL papain solution consisting of 20 units of papain in DMEM/F12, and 32.2 µg/mL DNase in a well of a 12-well plate (Sarstedt) for 60 min at 37°C. To support the dissociation, we shook the plate gently every 5 min for the first 30 min and carefully pipetted organoids up and down in wide-bore tips every 5 min for the remaining 30 min. Next, we passed the cell suspensions through a 70 µm cell strainer (Miltenyi), washed the cells with 0.1% BSA in PBS, and resuspended them in 1% BSA in PBS and immediately used them for scRNA-seq input.

scRNA-seq sample processing:

We used pooled samples from 8 organoids per organoid type as described above (FBOs, ETMR-, and ATRT-SHH-FBOs). For the cell lines, we used one well of a 6-well plate for ETMR cells, and a T25 flask for ATRT-SHH cells. For each cohort, 10,000 cells were used as input for further processing.

We processed single-cell suspensions using established protocols by 10x Genomics, using Chromium Single Cell 3' Gel Bead Kit v3.1 (PN-1000121, 10x Genomics, Pleasanton, CA, USA) and a Chromium Next GEM Chip G Single Cell Kit (PN-1000127, 10x Genomics). Briefly, after cell dissociation of the samples, we loaded 10,000 cells onto a chip to generate gel beads in emulsion (GEMs) using a Chromium single-cell controller (10x Genomics), followed by reverse transcription in individual GEMs. We used the Library Bead Kit (PN-1000157) and Chromium i7 Multiplex Kit (PN-120262) to generate single-indexed single-cell libraries for Illumina sequencing (sample KK21-H-069) and the Library Construction Kit (PN-1000190) and Dual Index Kit TT Set A (PN-1000123) for double-indexed libraries (all other samples). We determined the quality, purity, size, and concentrations of cDNA libraries using a Tapestation 2000 (Agilent Technologies, Santa Clara, CA, USA). Samples were sequenced on a NextSeq 2000 instrument (high-throughput kit, 100 cycles) at the Genomics Core Facility (University of Münster, Germany). Sample specifications and quality control results can be found in **Supplementary Table S6**.

Fixed single-nuclei RNA (snRNA)-sequencing

Sample Preparation – Cell Lines: ETMR and ATRT cell lines were cultured in their standard media (see Section "Cell culture") and dissociated into single cells using 1 mL Accutase (37°C, 3–5 min). Following dissociation, cells were resuspended in culture medium for counting. Cell suspensions containing 1–2 million cells were centrifuged at 300 ×*g* for 5 min at 4°C, and the pellets were resuspended in 1 mL Fixation Buffer (4% formaldehyde in 1× Fix & Perm Buffer; 10x Genomics, PN-2000517). Fixation was performed at 4°C for 16–24 h. Fixed cells were then centrifuged at 950 ×*g* for 5 min at room temperature and quenched with 1 mL of Quenching Buffer (1× Quench Buffer; 10x Genomics, PN-2000516) prior to library preparation.

Sample Preparation – FFPE Tissues: FFPE human ETMR and ATRT tumor tissues were processed using the Chromium Next GEM Single Cell Fixed RNA Sample Preparation Kit (10x Genomics, PN-1000414, protocol CG000632_RevD). Three 25-µm-thick sections per sample were deparaffinized, rehydrated, and dissociated into nuclei using the gentleMACS Octo Dissociator with FFPE-specific protocols (Miltenyi Biotec, Bergisch Gladbach, Germany, 130-096-427) and a freshly prepared Dissociation Enzyme Mix containing Liberase TH (Millipore Sigma, Burlington, MA, USA, 5401151001). After filtration, single-nuclei suspensions were resuspended in 1 mL of Quenching Buffer prior to library preparation.

Library Preparation: For both fixed cell lines and FFPE-derived samples, nuclei suspensions were supplemented with 0.1 volumes of Enhancer (10x Genomics, PN-2000482) and 10% glycerol for long-term storage. Prior to library construction, samples were thawed at room temperature, centrifuged at 950 ×*g* for 5 min at room temperature, and resuspended in 1 mL of 0.5× PBS with 0.02% BSA for nuclei counting using the Countess 3 Automated Cell Counter (Thermo Fisher Scientific, 16842556). For each sample, one million nuclei were hybridized with human transcriptome probes for 20 h at 42°C. Multiplexed libraries were prepared according to protocol CG000527_RevE (10x Genomics). Barcoded nuclei were pooled, washed, and loaded onto a microfluidic chip with a target recovery of 80,000 cells per 16-plex library.

Bioinformatic analysis of sc/snRNA-seq data:

All original organoid and cell line scRNA-seq samples were analyzed with 10x Genomics' CellRanger pipeline v6.0.2 [17]. After converting the raw 10x input data to FASTQ format with CellRanger's mkfastq routine, the data was aligned to the respective reference transcriptomes hg38 and mm10 v2020-A with CellRanger count using default values. Resulting count tables were imported into R with Seurat's Read10x function. For the published single-cell and single-nuclei datasets from Herring et al. [18], Polioudakis et al. [19], and Xu et al. [20], raw count matrices per sample were collected and combined with the original data for an integrated Seurat workflow (v4.0.5) [21].

For all sc/snRNA-seq samples, Seurat objects were created based on the following strategy: Seurat filters were set to a minimum of 3 cells, a minimum feature number of 50, and a maximum of 25% mitochondrial genes per cell. Potential doublets were

assessed with Seurat's nCount_RNA value; all outlier cells with very high-count values were removed from the dataset. The remaining data was normalized and integrated following Seurat's SCTransform (SCT) workflow for all datasets except the murine ETMR dataset. First, Seurat's SCT routine was applied to each sample of a chosen set's sample list. Three thousand integration features were selected for each list, and the SCT integration was prepared based on the transformed sample list and selected anchor features. Subsequently, integration anchors were calculated using the normalization method SCT and reciprocal principal component analysis (PCA) as a reduction method. The data was then integrated with the previously calculated anchors, SCT as normalization method, default dimensions for the anchor weighting process, and a k.weight parameter of 50. PCA and uniform manifold approximation and projection plots (UMAP) were created for each dataset based on Seurat's RunPCA and RunUMAP functionality. Afterwards, dimension plots and feature plots were created using the homonymous Seurat functions in order to visualize the distribution of RNA count values and sample annotation. Details about the different datasets, the included samples and referring figures can be found in **Supplementary Tables S7-S8**.

In preparation for Seurat's clustering function, sets of nearest neighbors between cells were determined with Seurat's FindNeighbors function. Resolution parameters of 0.5 were employed to create initial clusterings, and cell type annotations were performed by domain experts using the expression of marker genes per cluster. In the case of ambiguous clusters that showed a clear split into subpopulations, further subclusterings were conducted. For the murine ETMR dataset, a Harmony-based workflow (v0.1.0) [22] was used instead to account for the dataset's heterogeneity. Here, all samples were merged, normalized, and scaled before Harmony was used to integrate the data. For the integration of tumor cells from primary tumor tissue, TBO, and tumor spheres, tumor cells were isolated based on cluster identity. For the extraction of human tumor cells from TBO samples, sex-determining genes were used. Specifically, for male human ETMR cells (originating from BT-183) the expression of Y-chromosomal genes (USP9Y or RPS4Y1) over a threshold of 0.1 on the mean log-normalized Seurat expression values was used. ATRT cells were extracted based on a combination of SMARCB1 expression < 1 (SMARCB1-loss as a hallmark for ATRT) and each sex-specific gene expression; for male human ATRT

cells (originating from CHLA-02), a threshold for the expression of Y-chromosomal gene RPS4Y1 ≥ 1 was applied, and, for female human ATRT cells (originating from SHH-310FHTC) a X-inactive specific transcript (XIST)-expression of ≥ 1 (a gene being expressed to inactivate the second X-chromosome in female cells, with the exception of female pluripotent stem cells; like all hiPSC-derived cells [23]). Murine ETMR cells were extracted based on the murine genome of the cells. A list of all sc/snRNA-seq samples allocated to the respective figures and datasets can be found in **Supplementary Tables S5, S7-S8**.

Feature plots, UMAP visualizations, and dot plots were created with Seurat functions and ggplot2 [12] for all chosen datasets, subsets, and marker genes, independent of the clustering strategy. Cell cycle plots were generated using published signatures [24]. Heatmaps were visualized using the R package pheatmap [25]. To identify differentially expressed marker genes between clusters or conditions, we used Seurat's findMarkers with a two-sided MAST statistical test. Cell annotation was performed based on marker gene expression (**Supplementary Table S9**).

Drug screening results were validated using a computational prediction model from Sinha et al. [26]. Raw counts of fresh single-cell and fixed single-nucleus RNAseq data of hETMR cells were used as the inputs for this model, predicting drug viabilities for all available 44 drug targets separately for the two datasets. All functions of the implemented pipeline were run with default parameters.

References:

1. Neumann JE, Wefers AK, Lambo S, Bianchi E, Bockstaller M, Dorostkar MM, et al. A mouse model for embryonal tumors with multilayered rosettes uncovers the therapeutic potential of Sonic-hedgehog inhibitors. Nat Med. 2017;23(10):1191–202.

2. de Faria FW, Walter C, Interlandi M, Melcher V, Riedel N, Graf M, et al. ETMR-05. Single-cell transcriptomics of ETMR reveals developmental cellular programs and tumor-pericyte communications in the microenvironment. Neuro Oncol. 2022 Jun;24(Supplement_1):i50–i50.

3. Spence T, Perotti C, Sin-Chan P, Picard D, Wu W, Singh A, et al. A novel

C19MC amplified cell line links Lin28/let-7 to mTOR signaling in embryonal tumor with multilayered rosettes. Neuro Oncol. 2014 Jan 1;16(1):62–71.

4. Reinhardt P, Glatza M, Hemmer K, Tsytsyura Y, Thiel CS, Höing S, et al. Derivation and Expansion Using Only Small Molecules of Human Neural Progenitors for Neurodegenerative Disease Modeling. PLoS One. 2013 Mar;8(3):e59252.

5. Spence T, Perotti C, Sin-Chan P, Picard D, Wu W, Singh A, et al. A novel C19MC amplified cell line links Lin28/let-7 to mTOR signaling in embryonal tumor with multilayered rosettes. Neuro Oncol. 2014 Jan 1;16(1):62–71.

6. Wu S, Ying G, Wu Q, Capecchi MR. Toward simpler and faster genome-wide mutagenesis in mice. Nat Genet. 2007;39(7):922–30.

7. Renner H, Grabos M, Becker KJ, Kagermeier TE, Wu J, Otto M, et al. A fully automated high-throughput workflow for 3d-based chemical screening in human midbrain organoids. Elife. 2020 Oct 1;9:1–39.

8. Sloan SA, Andersen J, Pașca AM, Birey F, Pașca SP. Generation and assembly of human brain region–specific three-dimensional cultures. Nat Protoc. 2018 Sep 1;13(9):2062–85.

9. Schmidt LH, Brand C, Stucke-Ring J, Schliemann C, Kessler T, Harrach S, et al. Potential therapeutic impact of CD13 expression in non-small cell lung cancer. PLoS One. 2017;12(6):1–16.

10. Renner H, Otto M, Grabos M, Schöler HR, Bruder JM. Fluorescence-based single-cell analysis of whole-mount-stained and cleared microtissues and organoids for high throughput screening. Bio-protocol. 2021 Jun 20;11(12).

11. Renner H, Becker KJ, Kagermeier TE, Grabos M, Eliat F, Günther P, et al. Cell-Type-Specific High Throughput Toxicity Testing in Human Midbrain Organoids. Front Mol Neurosci. 2021 Jul 15;14.

12. Wickham H. ggplot2: Elegant Graphics for Data Analysis. Springer-Verlag New York. 2016;ISBN 978-3.

13. Team RC. R: A language and environment for statistical computing. R Found Stat Comput Vienna, Austria. 2022;(URL https://www.R-project.org/.).

14. Mao J, Ligon KL, Rakhlin EY, Thayer SP, Bronson RT, Rowitch D, et al. A Novel Somatic Mouse Model to Survey Tumorigenic Potential Applied to the Hedgehog Pathway. Cancer Res. 2006 Oct;66(20):10171–8.

15. Zhuo L, Theis M, Alvarez-Maya I, Brenner M, Willecke K, Messing A. hGFAP-cre transgenic mice for manipulation of glial and neuronal function in vivo. genesis. 2001 Oct;31(2):85–94.

16. Harada N, Tamai Y, Ishikawa T, Sauer B, Takaku K, Oshima M, et al. Intestinal polyposis in mice with a dominant stable mutation of the β-catenin gene. EMBO J. 1999 Nov 1;18(21):5931–42.

17. Zheng GXY, Terry JM, Belgrader P, Ryvkin P, Bent ZW, Wilson R, et al. Massively parallel digital transcriptional profiling of single cells. Nat Commun. 2017;8:1–12.

18. Herring CA, Simmons RK, Freytag S, Poppe D, Moffet JJD, Pflueger J, et al. Human prefrontal cortex gene regulatory dynamics from gestation to adulthood at single-cell resolution. Cell. 2022 Nov;185(23):4428-4447.e28.

19. Polioudakis D, de la Torre-Ubieta L, Langerman J, Elkins AG, Shi X, Stein JL, et al. A Single-Cell Transcriptomic Atlas of Human Neocortical Development during Mid-gestation. Neuron. 2019 Sep 4;103(5):785-801.e8.

20. Xu Y, Zhang T, Zhou Q, Hu M, Qi Y, Xue Y, et al. A single-cell transcriptome atlas profiles early organogenesis in human embryos. Nat Cell Biol. 2023;25(4):604–15.

21. Hao Y, Hao S, Andersen-Nissen E, Mauck WM, Zheng S, Butler A, et al. Integrated analysis of multimodal single-cell data. Cell. 2021;184(13):3573-3587.e29.

22. Korsunsky I, Millard N, Fan J, Slowikowski K, Zhang F, Wei K, et al. Fast, sensitive and accurate integration of single-cell data with Harmony. Nat Methods. 2019;16(12):1289–96.

23. Cloutier M, Kumar S, Buttigieg E, Keller L, Lee B, Williams A, et al. Preventing erosion of X-chromosome inactivation in human embryonic stem cells. Nat Commun. 2022;13(1):2516.

24. Tirosh I, Izar B, Prakadan SM, Wadsworth MH, Treacy D, Trombetta JJ, et al. Dissecting the multicellular ecosystem of metastatic melanoma by single-cell RNA-seq. Science (80-). 2016 Apr;352(6282):189–96.

25. Kolde R. Pheatmap: pretty heatmaps. R Packag version. 2019;1(2):726.

26. Sinha S, Vegesna R, Mukherjee S, Kammula A V, Dhruba SR, Wu W, et al. PERCEPTION predicts patient response and resistance to treatment using single-cell transcriptomics of their tumors. Nat Cancer. 2024;5(6):938–52.

Supplementary Figures

Supplementary Figure S1: FBOs differentiated over time and developed diverse neuronal and glial cell types.

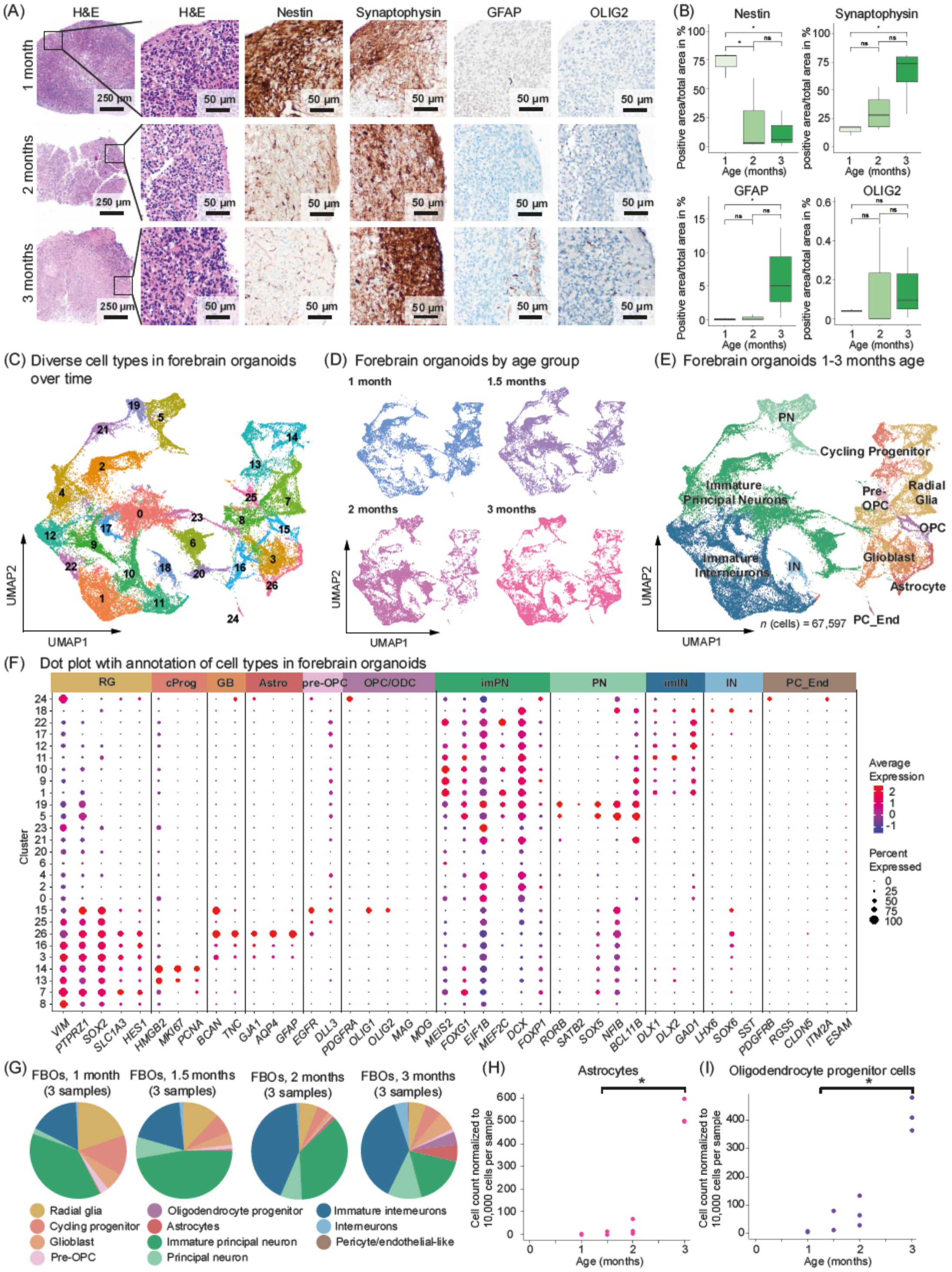

Supplementary Figure S1: FBOs differentiated over time and developed diverse neuronal and glial cell types. FBOs contained a plethora of distinct cell types and matured with culture age. (A) Immunohistological stainings from serial sections of FBOs at 1, 2, and 3 months show one representative FBO each per label and age

condition (each block contained > 40 FBOs). One-month-old FBOs were Nestin^{+}, Synaptophysin^{+}, GFAP^{-} and OLIG2^{-}. With time FBO became Nestin^{-}, stayed Synaptophysin^{+} and GFAP^{+} at 3 months. At 2 months single cells appeared OLIG2^{+}. (B) IHC quantification revealed FBO maturation over time. Boxplots depicting data for $n = 3$ FBOs per age group and staining. Two-sided Wilcoxon rank sum test, * P-value ≤ 0.05, ns: non-significant. (C-E) UMAP including 65,597 cells of scRNA-seq from 96 FBOs (1, 1.5, 2, and 3 months) ($n = 3$ samples per age; one sample consists of 8 pooled FBOs) colored by unsupervised clustering (C), split and colored by age group (D), and by cell type (E). (F) Dot Plot depicting the fetal forebrain cell type marker gene expression in FBO. The size of the dots represents the percentage of cells expressing the gene per cluster of the FBO-dataset. (G) Cellular composition varied over time, depicting FBOs' progressive maturation. Pie charts represent cellular composition at four different organoid ages, colored by cell type. (H-I) FBOs gave rise to astrocytes and oligodendrocyte progenitor cells as they matured. Dot plots of early FBOs (1-1.5 months) and older FBOs (3 months) revealed a significant increase of astrocytes and OPCs over time. Cell numbers normalized to 10,000 cells per sample (1 dot represents one sample in each plot, and one sample = 8 pooled FBOs). Two-sided Wilcoxon rank sum test, * P-value < 0.05.

Abbreviations: FBO, forebrain organoid; FFPE, formalin-fixed paraffin embedded; H&E, Hematoxylin and eosin; GFAP, glial fibrillary acidic protein; OLIG2, Oligodendrocyte transcription factor 2; UMAP, Uniform Manifold Approximation and Projection; scRNA-seq, single cell RNA sequencing; RG, radial glia; cProg, cycling progenitors; GB, glioblasts; pre-OPC, precursor oligodendrocyte progenitor cells; Astro, astrocytes; imPN, immature principal neurons; PN, principal neurons; imIN, immature interneurons; IN, interneurons; OPC, oligodendrocyte progenitor cells; ODC, oligodendrocyte; PC_End, pericyte/endothelial-like cell.

Supplementary Figure S2: Cellular forebrain organoid composition recapitulated the cellular diversity and developmental trends of fetal brains.

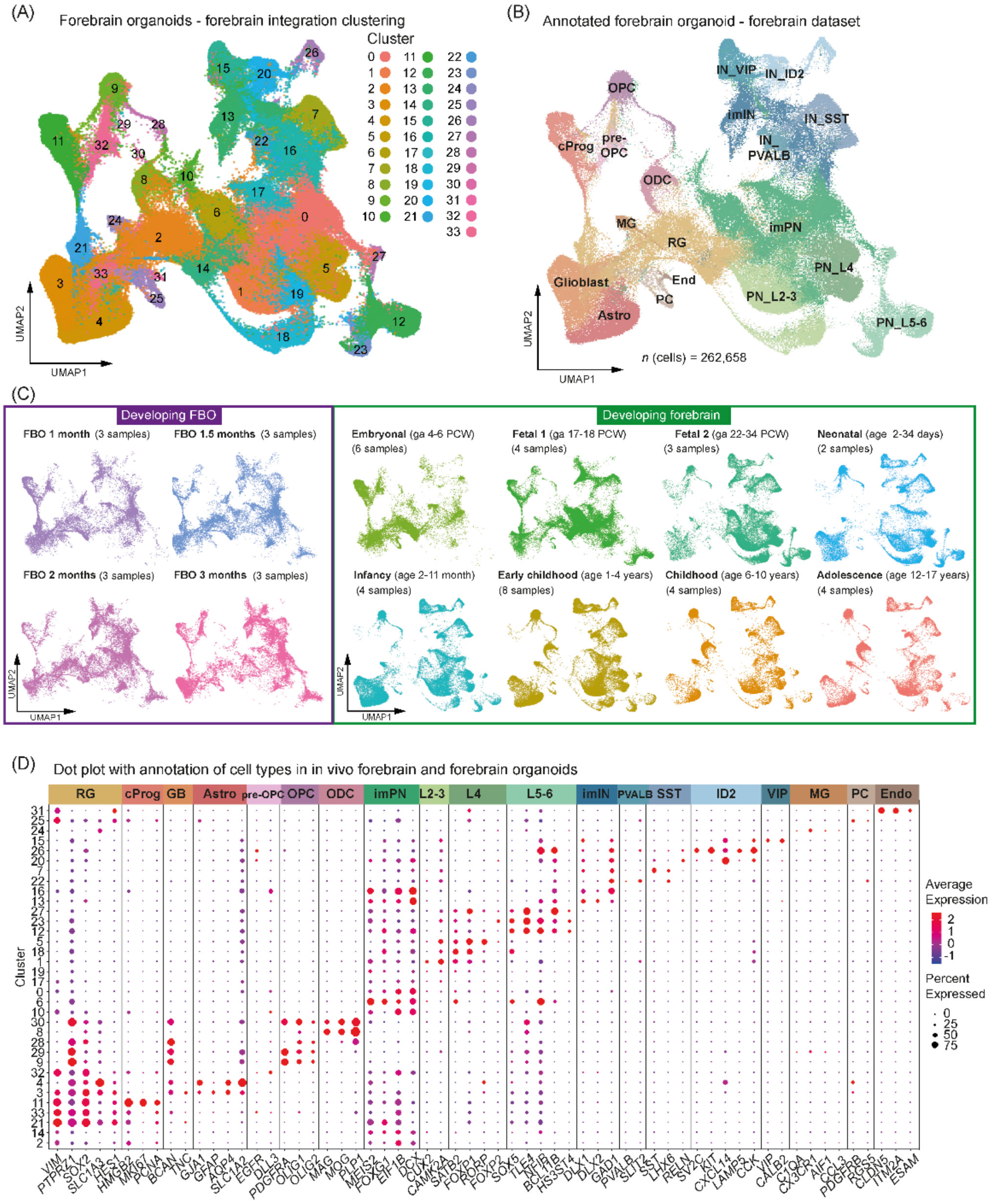

Supplementary Figure S2: Cellular forebrain organoid composition recapitulated the cellular diversity and developmental trends of fetal brains. (A-B) FBO expression profiles seamlessly integrated with datasets from early human neural development. UMAP containing 262,658 cell in total of 96 FBOs integrated with scRNA-seq and snRNA-seq from developing human forebrain samples; 7 embryonal scRNA-seq samples from Xu et al. [20] (ga 4-6 PCW), 4 fetal scRNA-seq samples from Polioudakis et al. [19] (ga 17-18 PCW; fetal1), 23 fetal to adolescent snRNA-seq samples from Herring et al. [18] (ga 22 PCW (fetal 2) - 17 years (adolescent), colored by unsupervised clustering (A) and cell type (B) (for more

details, **Supplementary Table S8**). (C) Detailed views of neural and FBO development across different age groups highlighted the emergence of specific clusters over time. UMAPs split into FBO age groups and *in vivo* developing forebrain age groups, displaying a large degree of overlap. (D) Dot plot illustrating average marker gene expression by cluster.

Abbreviations: FBO, forebrain organoid; UMAP, Uniform Manifold Approximation and Projection; scRNA-seq, single cell RNA sequencing; snRNA-seq, single nuclei RNA sequencing; ga, gestational age; PCW, postconceptional weeks; RG, radial glia; cProg, cycling progenitors; GB, glioblasts; Astro, astrocytes; OPC, oligodendrocyte progenitor cells; pre-OPC, precursor OPC; ODC, oligodendrocytes; imPN, immature principal neurons; L2-3, principal neurons layer 2-3; L4, principal neurons layer 4; L5-6, principal neurons layer 5-6; imIN, immature interneurons; PVALB, interneurons characterized by PVALB-expression; SST, interneurons characterized by SST-expression; ID2, interneurons characterized by ID2-expression; VIP, interneurons characterized by VIP-expression; MG, microglia; PC, pericytes; Endo, endothelial cells.

Supplementary Figure S3: FBOs shared developmental differentiation dynamics of early fetal development and contained similar cellular proportions as fetal brains.

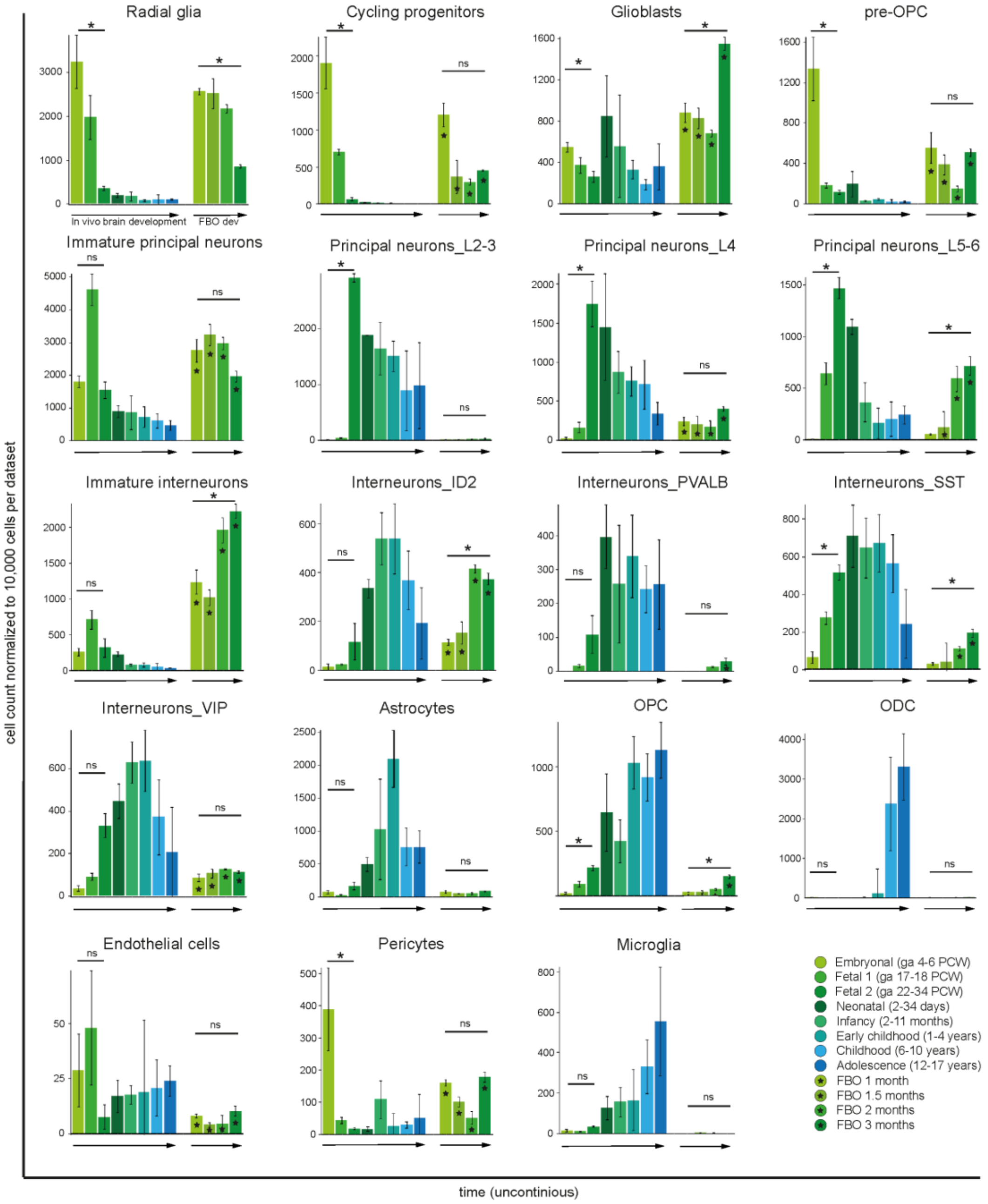

Supplementary Figure S3: FBOs shared developmental differentiation dynamics of early fetal development and contained similar cellular proportions as fetal brains. FBOs aged 1-3 months exhibited key cell populations and proportions similar to those found in embryonic and fetal samples. FBOs corresponded to the first three stages of *in vivo* development - embryonal (ga 4-6 PCW), fetal 1 (ga 17-18 PCW) and fetal 2 (ga 22-34 PCW), which are color-coded to match. FBO samples are marked with an asterisk. The bar graphs show the number of different cell types normalized to 10,000 cells per dataset in developing FBOs and human brains, categorized and

colored by age group and displayed in separate graphs for different cell types of interest. Two-sided Wilcoxon rank sum test performed only for the comparison of embryonal vs. fetal 2 and FBO 1 month and 3 months, * P-value ≤ 0.05, ns: non-significant.

Abbreviations: FBO, forebrain organoid; dev, development; principal neurons_Lx, principal neurons layer x; interneurons_xy, interneurons characterized by xy-expression; ODC, oligodendrocyte; OPC, oligodendrocyte progenitor cell; pre-OPC, precursor-OPC; ga, gestational week; PCW, post conceptual week; ns, non-significant.

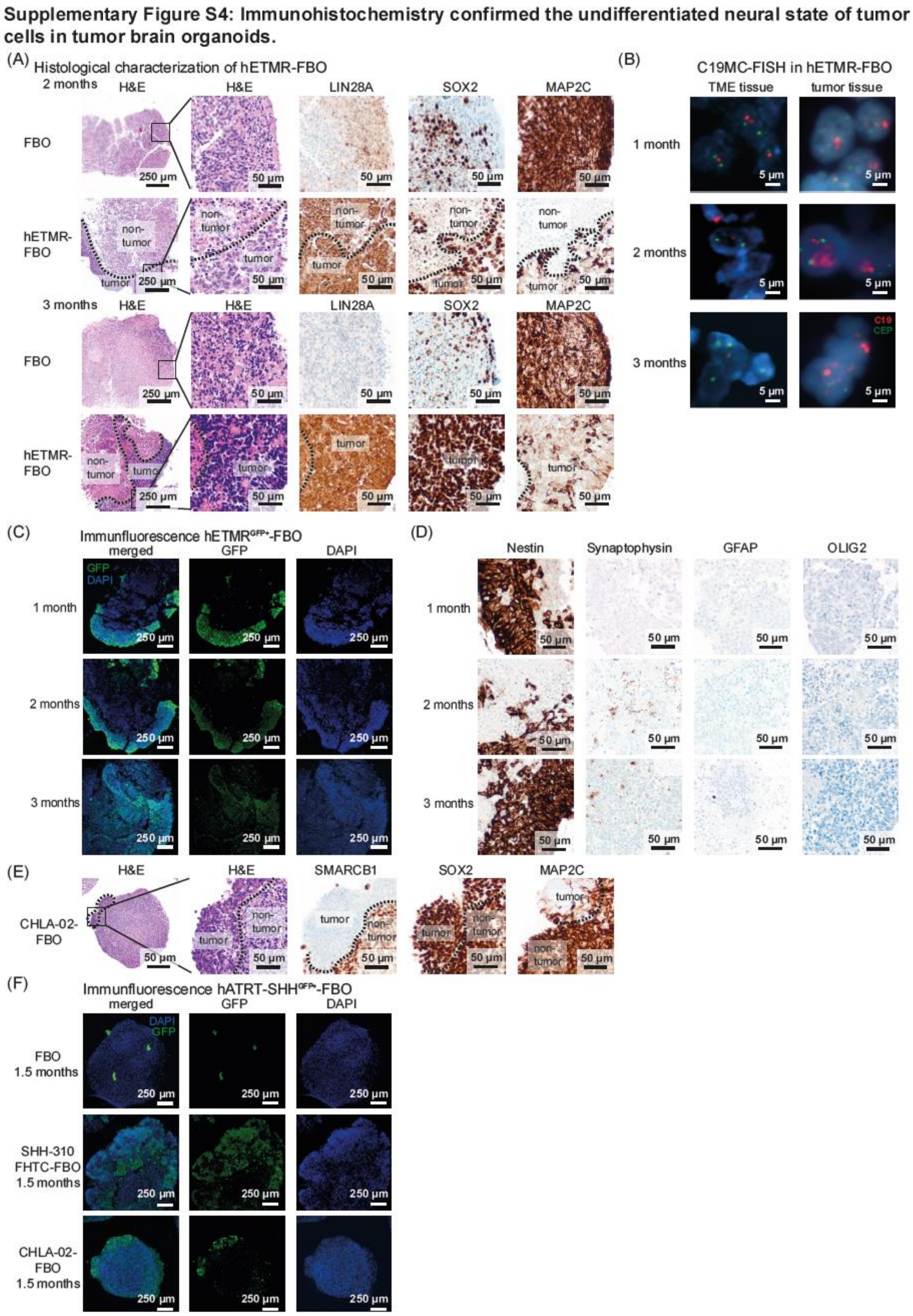

Supplementary Figure S4: Immunohistochemistry confirmed the undifferentiated neural state of tumor cells in tumor forebrain organoids. (A) Representative images of immunohistochemical staining of FBOs and hETMRGFP^{+}-FBOs at later ages (2 and 3 months) were generated from serial sections of a FFPE block containing > 40 FBOs. Boxes indicate areas shown in lower magnification. As a

hallmark for hETMR, the tumor cells were LIN28A$^+$, while the no-cancer-control FBOs became LIN28A$^-$ from 2 months onward. SOX2 in FBO was reduced to only a few positive cells at 2-3 months, while tumor cells in hETMRGFP$^+$-FBOs remained SOX2$^+$. 2-3 months old FBOs were MAP2C$^+$. In hETMRGFP$^+$-FBOs, single scattered cells showed MAP2C expression. (B) C19MC-Fluorescence in situ hybridization (FISH) confirmed amplification in tumor cells. (C) Immunofluorescence of hETMRGFP$^+$-FBOs at different ages (1, 2, and 3 months) confirmed tumor presence in TBO via GFP staining against hETMRGFP$^+$ cells, validating the location of tumor cells in cell-dense and highly LIN28A$^+$ areas in Figure 1D. (D) Additional stainings confirmed the immature neuronal phenotype of hETMR cells in TBOs, with Nestin$^+$, Synaptophysin$^-$, GFAP$^-$, and OLIG2$^-$. (E) Immunohistochemical stainings of CHLA-02GFP$^+$ at 1.5 months ($n > 40$ FBOs per FFPE block). H&E staining (magnified areas indicated by black boxes) showed hATRT cells with large nuclei and defined by SMARCB1-negativity. hATRT cells in the FBOs were largely SOX2$^+$, while MAP2C was positive only in a few cell bodies. (F) hATRT-SHHGFP$^+$-FBO tumor areas were confirmed via immunofluorescence (GFP), validating the location of tumor cells in cell-dense and SMARCB1$^-$ areas in Figure 1 E.

Abbreviations: FFPE, formalin-fixed paraffin-embedded; ETMR, embryonal tumor with multilayered rosettes; H&E, hematoxylin and eosin; SOX2, SRY-box transcription factor 2; GFP, green fluorescent protein; MAP2C, Microtubule-associated protein 2c; LIN28A, lin-28 homolog A; hETMRGFP$^+$-FBO, human ETMR-forebrain-organoid with GFP$^+$ tumor cells; FISH, fluorescence in situ hybridization; CEP, chromosome enumeration probes; TME, tumor microenvironment; TBO, tumor brain organoid; hATRT-SHHGFP$^+$-FBO, human ATRT-SHH-forebrain-organoid with GFP$^+$ tumor cells; GFAP, glial fibrillary acidic protein; OLIG2, oligodendrocyte transcription factor 2; SMARCB1, SWI/SNF-related matrix-associated actin-dependent regulator of chromatin subfamily B member 1; DAPI, 4′,6-Diamidin-2-phenylindol.

Supplementary Figure S5: Tumorsphere culture lead to the loss of primary tumor cell states and altered cell cycle dynamics.

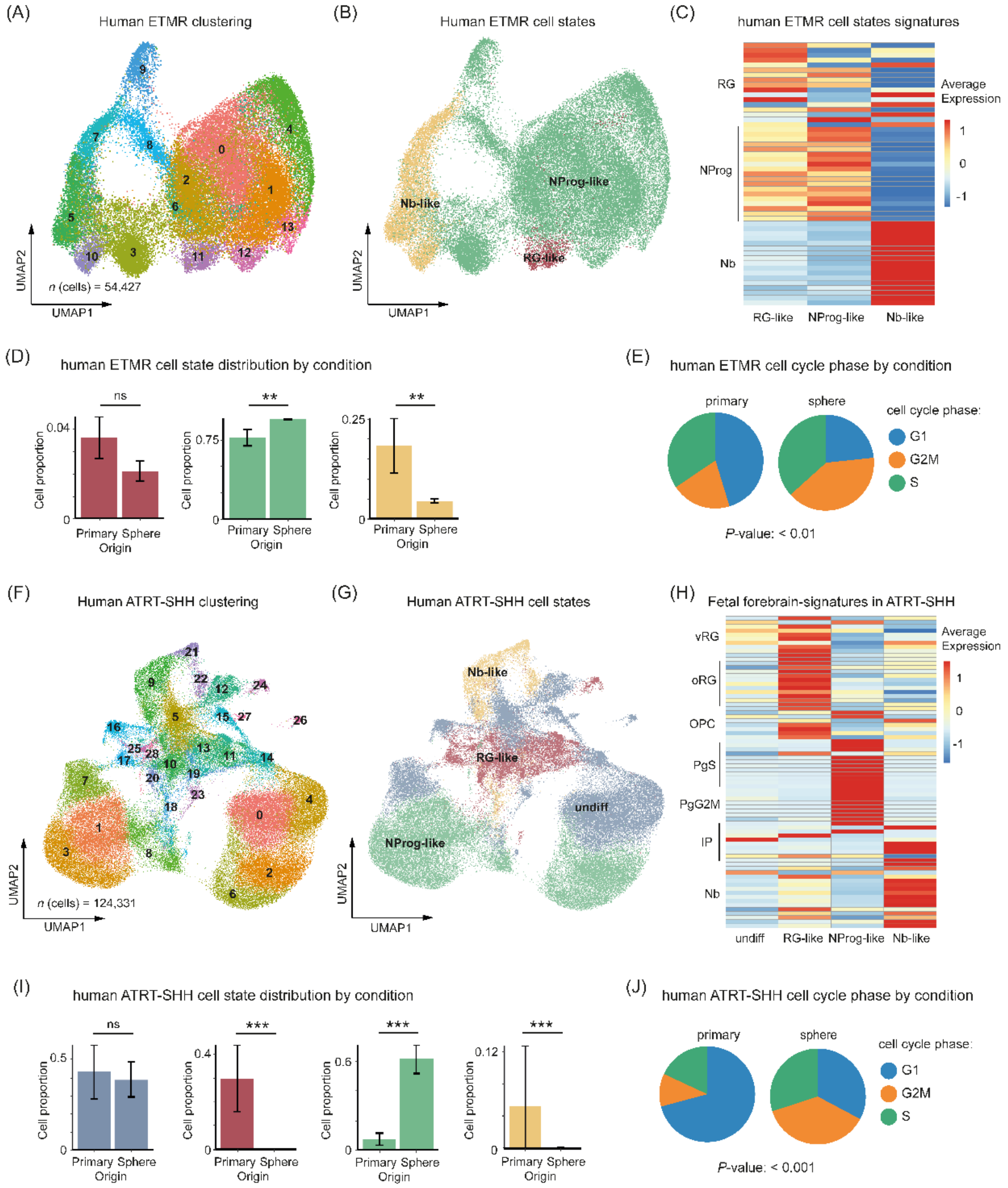

Supplementary Figure S5: Tumorsphere culture leads to the loss of primary tumor cell states and altered cell cycle dynamics. (A-B) UMAP of the integration of fixed snRNA-seq human tumor cells from primary ETMR and tumorspheres (*n* = 8 primary tumors, *n* = 4 BT-183 samples); colored by unsupervised clustering (A) or cell state (B). (C) Heatmap showing the expression of ETMR cell state signatures; color-coded by average gene expression. (D) Bar graph of ETMR cellular states by condition showed a different distribution in primary and spheres. Colored by cell state as in (B). (E) Pie charts showing the cell cycle distribution by condition. (F-G) UMAP

of the integration of fixed snRNA-seq human tumor cells from primary ATRT-SHH and tumorsphere cells ($n = 18$ primary tumors, $n = 10$ tumorsphere samples, each 2 per sphere line Chla02 and SHH310FHTC); colored by unsupervised clustering (F) or cell state (G). (H) Heatmap showing the expression of fetal forebrain signatures; color-coded by average gene expression. (I) Bar graph of ATRT-SHH cellular states by condition showed a different distribution in primary and spheres. Colored by cell state as in (G). (J) Pie charts showing the cell cycle distribution by condition. Two-sided Wilcoxon rank sum test, * P-value < 0.05, ** P-value < 0.01, *** P-value < 0.001.

Abbreviations: UMAP, Uniform Manifold Approximation and Projection; ETMR, embryonal tumor with multilayered rosettes; ATRT-SHH, atypical teratoid and rhabdoid tumor from the sonic hedgehog subgroup; hETMR/ATRT-SHH, human ETMR/ATRT-SHH; FBO, forebrain organoid; snRNA-seq, single nuclei RNA-sequencing; undiff, undifferentiated tumor cells; RG-like, radial glia like tumor cells; NProg-like, neuronal progenitor like tumor cells; Nb-like, neuroblast like tumor cells; RG, radial glia; NProg, neuronal progenitor; Nb, neuroblast; ns, non-significant; vRG, ventral radial glia; oRG, outer radial glia; OPC, oligodendrocyte progenitor cells; PgS, progenitors in S-phase; PgG2M, progenitors in G2-M-phase; IP, intermediate progenitor; Nb, neuroblasts.

Supplementary Figure S6: Tumor cells in TBO displayed hallmarks of stalled neuronal development.

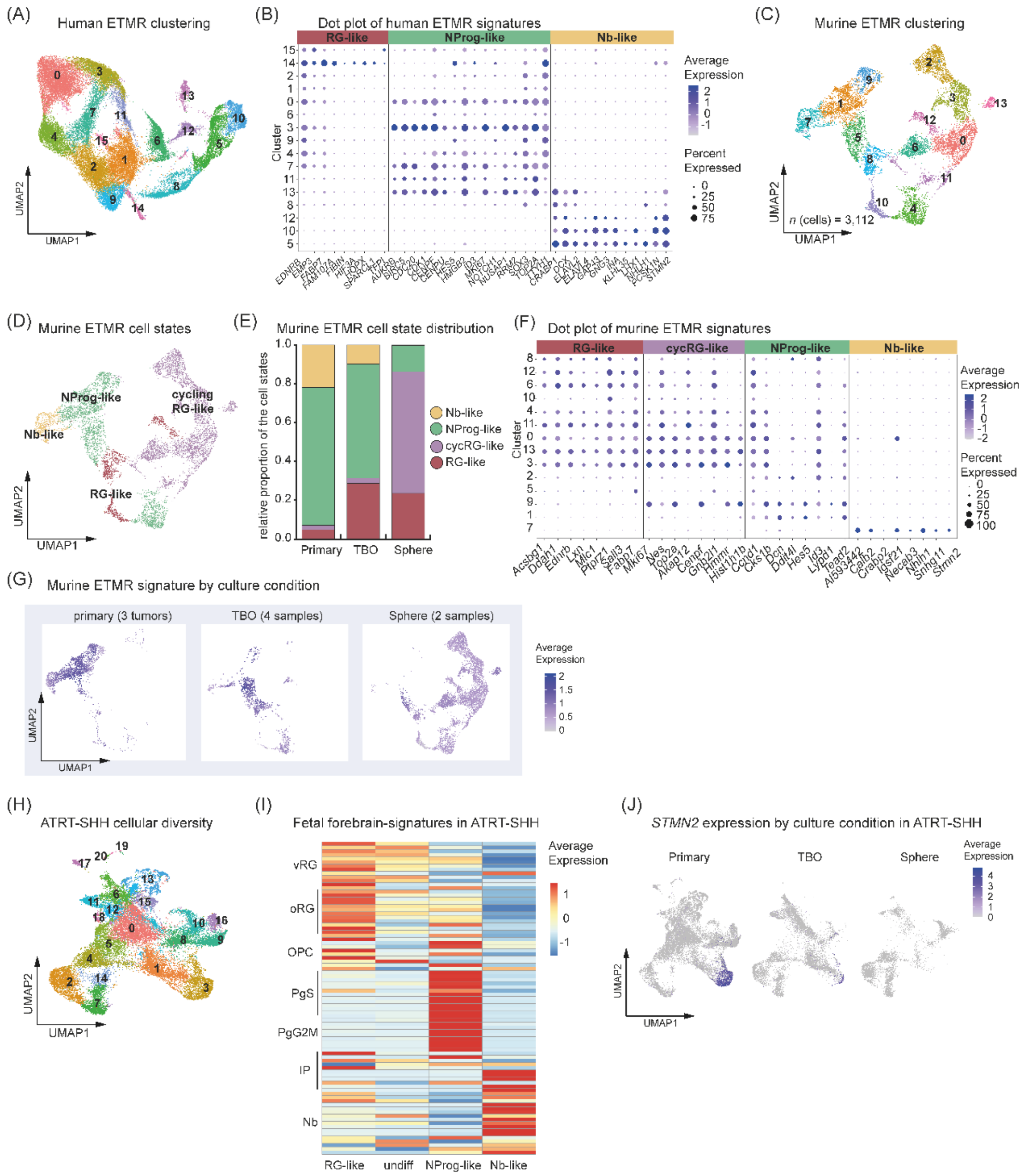

Supplementary Figure S6: Tumor cells in TBO displayed hallmarks of stalled neuronal development. (A) Integrated UMAP of tumor cells from primary human ETMR, hETMR-FBO and tumorsphere cells (n = 2 primary tumors, n = 6 TBO-samples 1 month, n = 2 BT-183 samples); colored by unsupervised clustering. (B) Dot plot of the human ETMR cell state signatures colored by average gene expression. The size of the dots represents the percentage of cells expressing the gene per cluster. (C-D) Integrated UMAP of 3,112 tumor cells from primary murine ETMR, mETMR-FBO and tumorsphere cells (n = 3 primary tumors, n = 4 1-month TBO-samples, n = 2 mETMR tumorsphere samples); colored by unsupervised clustering (C) or mETMR

cell states (D). (E) Tumor cell culture method affected the tumor cell state with a high accumulation of cycling RG-like cells in tumorspheres. Illustrated in stacked bar graphs colored by mETMR-cell state. (F) Dot plot showing the mETMR cell state marker gene expression, colored by average expression. Dot size represents the percentage of cells expressing the gene per cluster. (G) Murine ETMR signature showed a high expression in primary and TBO cells, but low expression in tumorsphere cells. UMAP split by origin illustrates the average gene expression. (H) Integrated UMAP of tumor cells from primary hATRT-SHH, hATRT-SHH-FBO and tumorsphere cells (*n* = 4 primary tumors, *n* = 4 TBO-samples 1.5 months, *n* = 2 ATRT-SHH sphere cell lines); colored by unsupervised clustering. (I) Using fetal forebrain cell type gene signatures in a heatmap of the hATRT-SHH dataset revealed that tumor cells exhibit gene expression patterns similar to those found in healthy neuronal cell types, including radial glia (RG), neuronal progenitors (NProg), and neuroblasts (Nb). Color-coded by average gene expression. (J) STMN2 was highly expressed in hATRT-SHH primary and TBO cells within the Nb-like cluster but was absent in tumorsphere cells, illustrated in a UMAP, split by the origin of the tumor cells and color-coded by average gene expression of STMN2.

Abbreviations: TBO, tumor brain organoid; UMAP, Uniform Manifold Approximation and Projection; ETMR, embryonal tumor with multilayered rosettes; ATRT-SHH, atypical teratoid and rhabdoid tumor from the sonic hedgehog subgroup; hETMR/ATRT-SHH, human ETMR/ATRT-SHH; FBO, forebrain organoid; scRNA-seq, single cell RNA-sequencing; sphere, tumorspheres (cell lines); mETMR, murine ETMR; RG-like, radial glia like tumor cells; cycRG-like, cycling radial glia like cells; NProg-like, neuronal progenitor like tumor cells; Nb-like, neuroblast like tumor cells; undiff, undifferentiated tumor cells; STMN2, stathmin 2; CNS, central nervous system; vRG, ventral radial glia; oRG, outer radial glia; OPC, oligodendrocyte progenitor cells; PgS, progenitors in S-phase; PgG2M, progenitors in G2-M-phase; IP, intermediate progenitor; Nb, neuroblasts.

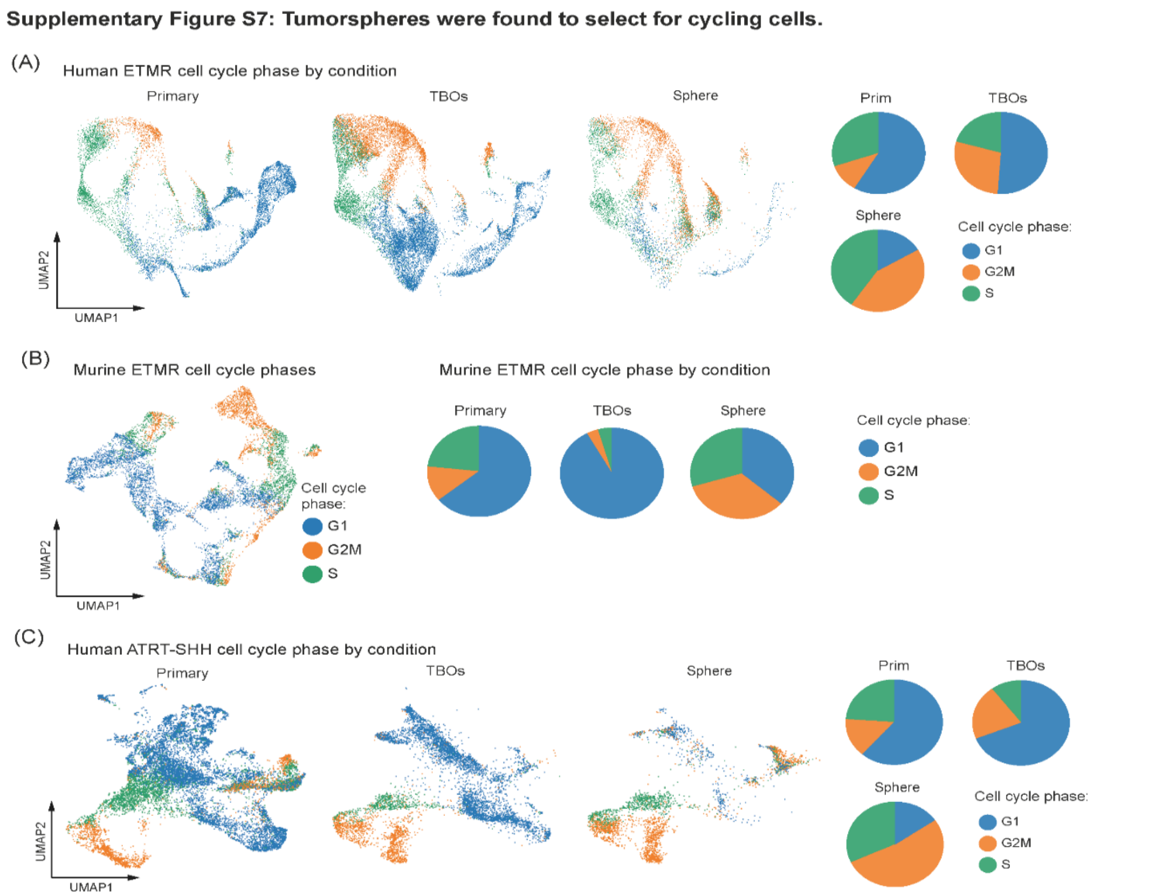

Supplementary Figure S7: Tumorspheres were found to select for cycling cells. (A) Cell cycle plots and pie charts for the human ETMR dataset split by origin of the tumor cells. hETMR showed a similar distribution of cycling cells in primary and TBO in contrast to tumorspheres which are > 80% cycling. Colored by cell cycle phase. (B) UMAP of the murine ETMR dataset colored by cell cycle phase. In contrast to 30% of cycling cells in primary murine ETMR tumors, tumorspheres showed an accumulation of cycling cells to 65% in comparison to 10% cycling cells in TBO (1 month) illustrated by pie charts colored by cell cycle phase. (C) Primary and hATRT-SHH cells from TBOs showed a division of the UMAP in cycling and non-cycling cells not present in tumorspheres, where cycling cells were spread over the whole UMAP. Primary and TBO hATRT-SHH cells showed a similar low number of cycling cells < 40% in contrast to tumorspheres with > 80% cycling cells, illustrated by pie charts. Colored by cell cycle phase.

Abbreviations: ETMR, embryonal tumor with multilayered rosettes; ATRT-SHH, atypical teratoid and rhabdoid tumor from the sonic hedgehog subgroup; hETMR/ATRT, human ETMR/ATRT-SHH; TBO, tumor brain organoid; UMAP, Uniform Manifold Approximation and Projection; spheres, tumorspheres (cell lines); prim, primary.

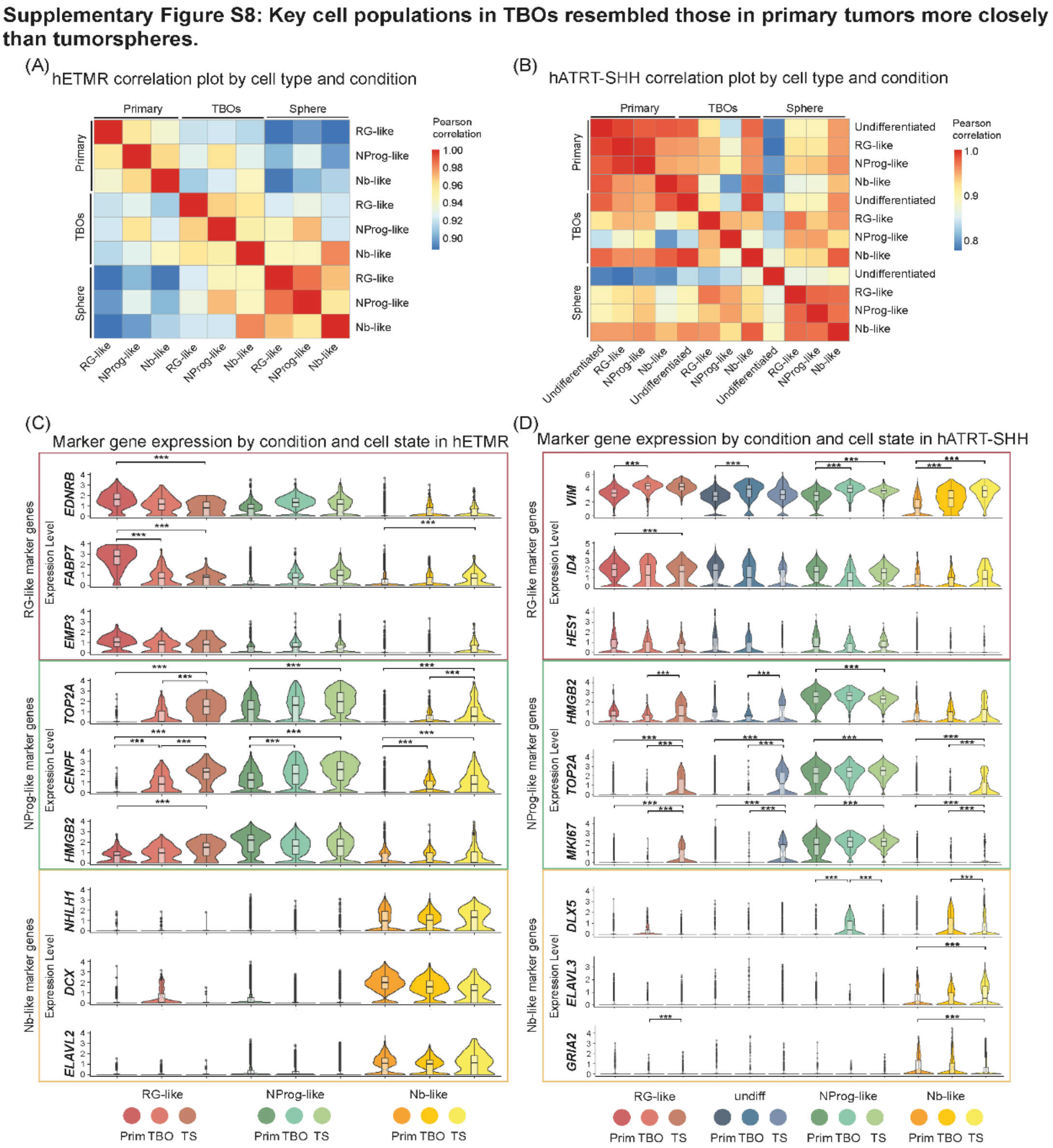

Supplementary Figure S8: Key cell populations in TBOs resembled those in primary tumors more closely than tumorspheres. (A-B) Pearson correlation plots illustrated the closer gene expression similarities between tumor cell states in TBOs and primary tumors compared to tumorspheres and primary tumors for both hETMR (A) and hATRT-SHH (B). Colored by Pearson correlation score. (C-D) Violin plots showing the marker gene expression in hETMR (C) or hATRT-SHH (D) for the main cell states, split into the three conditions. NProg-like maker genes were expressed across all cell states in tumorsphere cultures for hETMR and hATRT-SHH, whereas Nb-like gene expression was missing in hATRT-SHH tumorsphere cultures; *** *P*-value < 0.001 as taken from the differentially expressed gene list generated with Seurat´s “MAST” package. For clarity, significance indicators are only shown for log2 fold change > 1.0.

Abbreviations: UMAP, Uniform Manifold Approximation and Projection; ETMR, embryonal tumor with multilayered rosettes; ATRT-SHH, atypical teratoid and rhabdoid tumor from the sonic hedgehog subgroup; hETMR/ATRT-SHH, human ETMR/ATRT-SHH; prim, primary; spheres, tumorspheres (cell lines); RG-like, radial glia-like tumor cells; NProg-like, neuronal progenitor-like tumor cells; Nb-like, neuroblast-like tumor cells; undiff, undifferentiated tumor cells; TS, tumorsphere; TBO, tumor brain organoid.

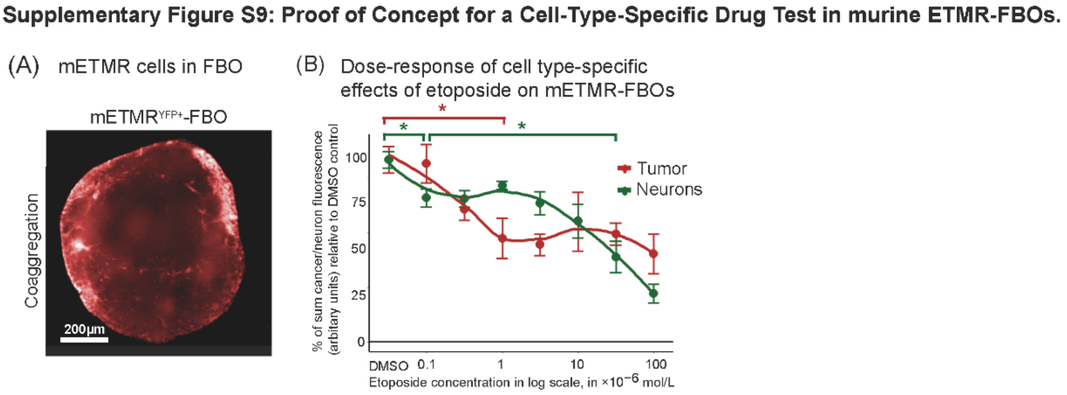

Supplementary Figure S9: Proof of Concept for a Cell-Type-Specific Drug Test in murine ETMR-FBOs. (A) Representative single confocal slice from high content imaging shows whole-mount immunostained and tissue-cleared mETMR-FBO. Murine ETMR (mETMRYFP$^+$) cells appeared broadly distributed in the FBO, with a tendency for the surface layers. (B) Dose-response testing for the tumor (YFP$^+$) and mature neuronal cells (MAP2$^+$) toxicity revealed an effective window for Etoposide treatment ($n \geq 4$ organoids per data point). All values are normalized to the mean of the sum brightness of the DMSO control. Dots represent the mean of the sum brightness of immunostained YFP$^+$ areas (tumor) and MAP2$^+$ signal (neurons) across the whole organoid in a maximum projection of all confocal planes. Error bars indicate the standard error of the mean. Asterisks indicate the first significant difference between the DMSO control and the first effective concentration in the tumor and neuronal compartments. A two-sided Wilcoxon rank sum test was used for the comparison between neuron-neuron and tumor-tumor data points, with a * P-value < 0.05 as significant.

Abbreviations: mETMR, murine embryonal tumor with multilayered rosettes; YFP, yellow fluorescent protein; mETMR-FBO, mETMR-forebrain organoid; MAP2, microtubule-associated protein 2; DMSO, dimethylsulfoxid.

Supplementary Figure S10: Compound screening in hETMR-FBO identified drugs with anti-tumor efficacy and low TME toxicity.

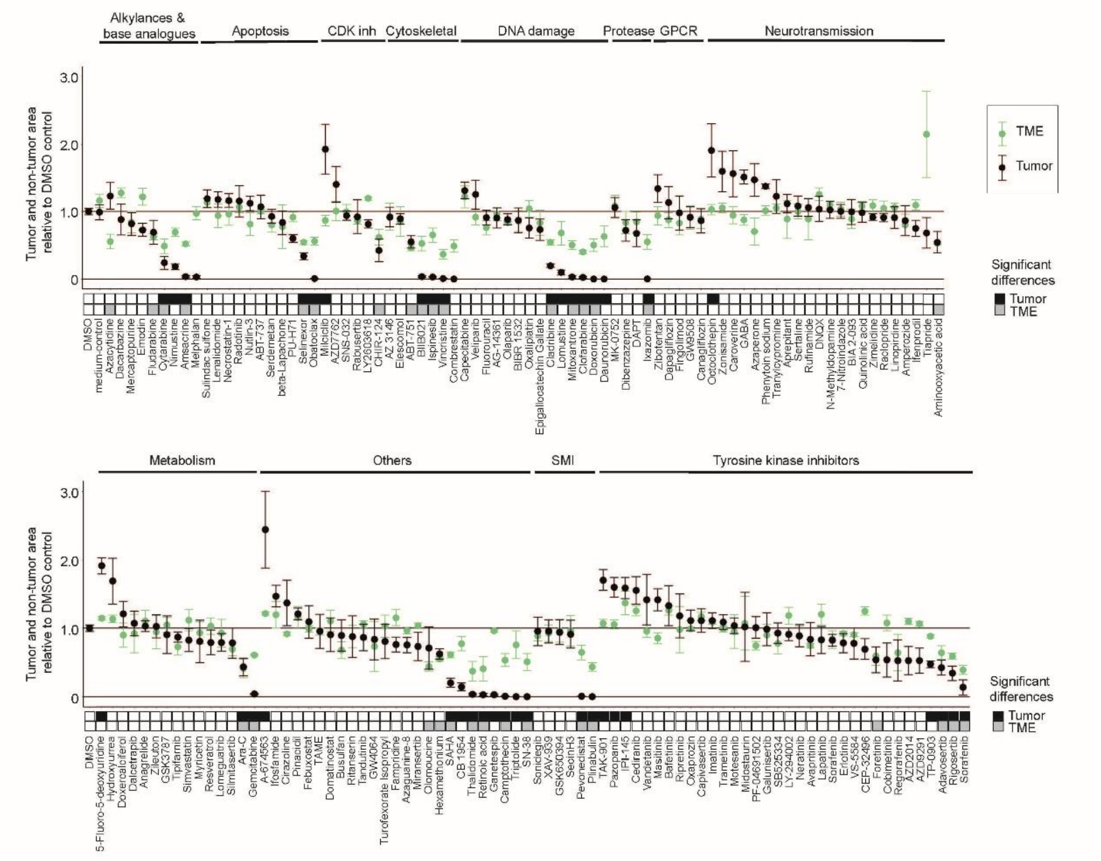

Supplementary Figure S10: Compound screening in hETMR-FBO identified drugs with anti-tumor efficacy and low TME toxicity. Compound treatment on hETMR-FBOs induced different changes in tumor cells (black dots) and TME areas (grey dots) in TBOs ($n \geq 4$ TBOs treated per drug). Dots represent the tumor/TME area segmented at each confocal z-plane, then summed across all confocal planes. Error bars indicate the standard error of the mean. All values are normalized to the mean of the summed tumor areas for the DMSO controls. Compounds are listed by their mechanism of action and sorted by anti-tumoral effect. Shaded boxes below the panel represent a significant change in the size of the tumor (black) and TME areas (grey) (*P*-value adj < 0.05 after multiple unpaired t-tests, and correction for multiple testing with Benjamini & Hochberg method). A white box represents no statistically significant change.

Abbreviations: hETMR, human embryonal tumor with multilayered rosettes; hETMR-FBOs, hETMR-forebrain organoid; TBO, tumor brain organoid; TME, tumor microenvironment; DMSO, dimethylsulfoxid; GPCR, G-protein coupled receptor; SMI, small molecule inhibitor; CDK, cyclin dependent kinase; inh, inhibitor.

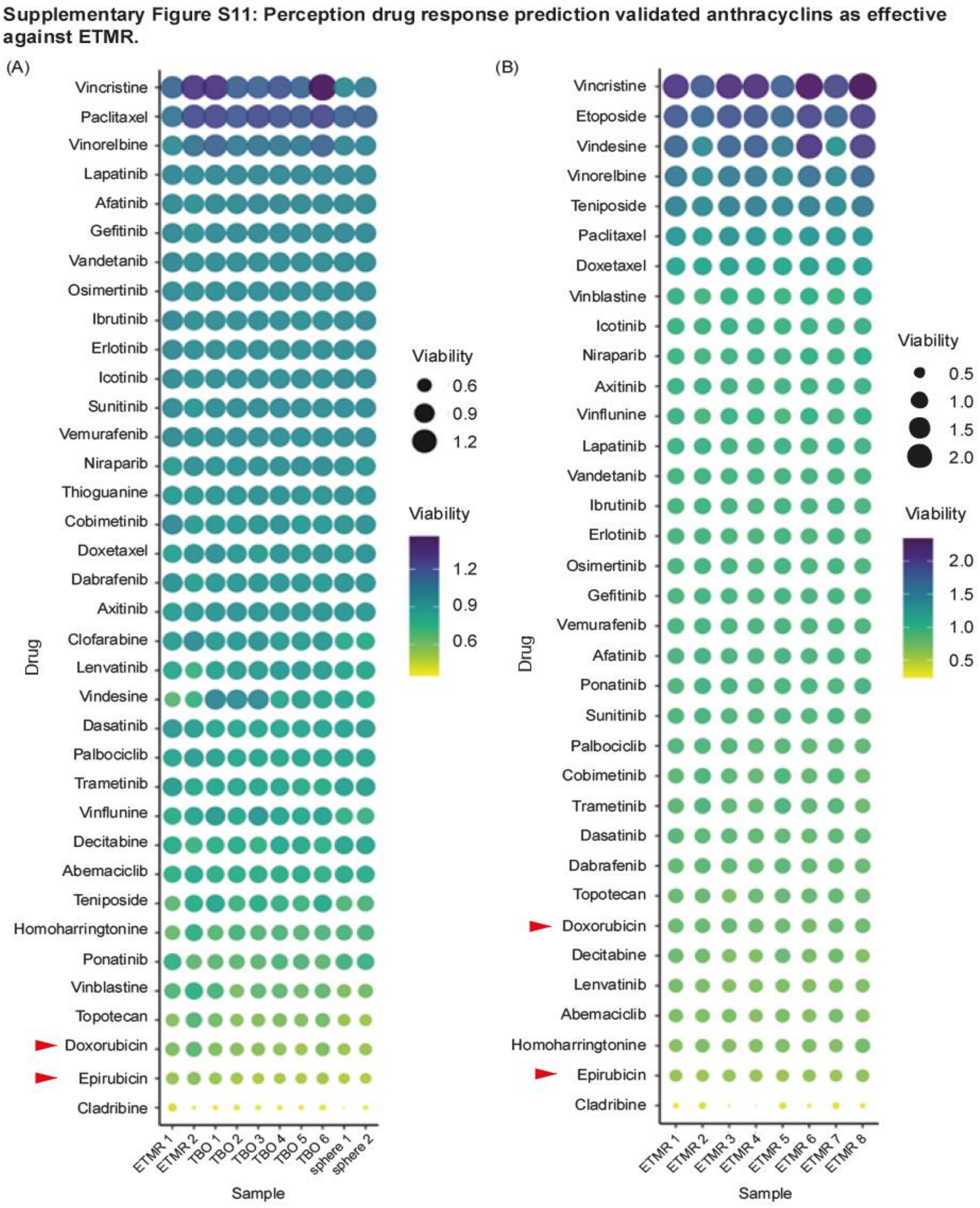

Supplementary Figure S11: Perception drug response prediction validated anthracyclines as effective against ETMR. (A-B) Dot plots showing the predicted drug response of ETMR cells based on scRNA-seq (A) and snRNA-seq (B) data using the PERCEPTION computational pipeline [26]. Ordered and colored by the viability of the drugs. The dot size represents the viability. Red arrows highlight the anthracyclines, epirubicin and doxorubicin.

Abbreviations: scRNA-seq, single-cell RNA sequencing; snRNA-seq, single-nuclei RNA sequencing; ETMR, embryonal tumor with multilayered rosettes; TBO, tumor brain organoid; sphere, tumorsphere.
